# Molecular Determinants of Juvenile Hormone Action as Revealed by 3D QSAR Analysis in *Drosophila*

**DOI:** 10.1371/journal.pone.0006001

**Published:** 2009-06-23

**Authors:** Denisa Liszeková, Maja Polakovičová, Milan Beňo, Robert Farkaš

**Affiliations:** 1 Laboratory of Developmental Genetics, Institute of Experimental Endocrinology, Slovak Academy of Sciences, Bratislava, Slovakia; 2 Department of Drug Chemical Theory, School of Pharmacy, Comenius University, Bratislava, Slovakia; 3 Department of Genetics, Graduate Programme, School of Science, Comenius University, Bratislava, Slovakia; Michigan State University, United States of America

## Abstract

**Background:**

Postembryonic development, including metamorphosis, of many animals is under control of hormones. In *Drosophila* and other insects these developmental transitions are regulated by the coordinate action of two principal hormones, the steroid ecdysone and the sesquiterpenoid juvenile hormone (JH). While the mode of ecdysone action is relatively well understood, the molecular mode of JH action remains elusive.

**Methodology/Principal Findings:**

To gain more insights into the molecular mechanism of JH action, we have tested the biological activity of 86 structurally diverse JH agonists in *Drosophila melanogaster*. The results were evaluated using 3D QSAR analyses involving CoMFA and CoMSIA procedures. Using this approach we have generated both computer-aided and species-specific pharmacophore fingerprints of JH and its agonists, which revealed that the most active compounds must possess an electronegative atom (oxygen or nitrogen) at both ends of the molecule. When either of these electronegative atoms are replaced by carbon or the distance between them is shorter than 11.5 Å or longer than 13.5 Å, their biological activity is dramatically decreased. The presence of an electron-deficient moiety in the middle of the JH agonist is also essential for high activity.

**Conclusions/Significance:**

The information from 3D QSAR provides guidelines and mechanistic scope for identification of steric and electrostatic properties as well as donor and acceptor hydrogen-bonding that are important features of the ligand-binding cavity of a JH target protein. In order to refine the pharmacophore analysis and evaluate the outcomes of the CoMFA and CoMSIA study we used pseudoreceptor modeling software PrGen to generate a putative binding site surrogate that is composed of eight amino acid residues corresponding to the defined molecular interactions.

## Introduction

Many aspects of the postembryonic development and reproduction of *Drosophila* and other insects are regulated by the coordinate action of two principal hormones, the steroid 20-hydroxyecdysone (hereafter referred to as ecdysone) and sesquiterpenoid juvenile hormone (JH). Mode of ecdysone action is relatively well known, in part due to extensive research in vertebrate steroid endocrinology that supported research on ecdysone action in *Drosophila* and other insects [1], [2]. On the other hand, the molecular mechanism(s) underlying JH action remain enigmatic; our incomplete understanding of JH action is not due to a lack of effort [3]–[6], but rather originates from the uniqueness of JH as a hormone [7].

The chemical nature of JH was suggested after JH activity was identified as farnesol derivatives in *Tenebrio molitor* excrements [8]. In the late 1960s, the first of several JH homologues were chemically identified [9]. Most insects have so called JH-III (epoxy farnesoic acid methyl ester) as natural juvenile hormone [10]. The hormone plays critical roles in a rich array of processes, including development, reproduction, behavior, pheromone production, adult diapause, polyphenism, and morph and caste determination [11], [12]. Perhaps most intriguing are the functions of JH associated with metamorphosis and reproduction [13]–[15].

Though Gilbert *et al*. [16] in one of his reviews wrote, in all of endocrinology there is no more wondrous name for a hormone than the insect juvenile hormone,“ its molecular and cellular modes of action are yet to be understood. Many laboratories and agrochemical companies have synthesized over 4000 analogs (agonists) of JH and these have been tested on hundreds of insect species as potential insecticides [17]–[22]. In terms of chemical synthesis, no other hormone in the animal kingdom or human medicine led to the production of so many agonists. Still, none of JH analogs have been used as widely as less specific and sometime toxic insecticides (*e.g.* chlorinated hydrocarbons, organophosphates, phenothiazines, pyrethroids, neonicotinoids, dinitrophenol insecticides, pyrazole insecticides, chitin synthesis inhibitors *etc*). *Drosophila*, though perhaps the best known representative of cyclorrhaphous diptera and an ideal genetic model organism, was mostly ignored in this research effort, as it is not a pest. Only a limited number of JH analogs (also known as juvenoids) were tested in *Drosophila* [23]–[27] and their structure-activity relationships were never evaluated.

In the genomic era, studies utilizing *Drosophila* offer considerable hope to understand the molecular mechanism of JH action and to identify the JH receptor, and thus to explain the plethora of data accumulated in the past four decades on JH. Here, we report results that characterize the precise pharmacological relationships of JH and its putative target in *Drosophila*. Such molecular analyses has proven to be a very useful tool in elucidating the molecular action of many compounds, including drugs, and predicting new pharmaceutically successful compounds. Examples include steroid agonists and antagonists [28], acetylcholinesterase inhibitors for Alzheimer symptoms treatment [29], dopamine receptor agonists [30], antimalarial drugs [31] and multidrug resistance modulators [32]. To gain insight into the molecular mechanism of JH action we first tested the biological activity of 86 JH agonists in a *Drosophila* morphogenetic assay. We then related these data to 3D QSAR–CoMFA and CoMSIA analyses (comparative molecular field analysis, comparative molecular similarity indices analysis, respectively). The widely used CoMFA calculates steric and electrostatic properties according to Lennard-Jones and Coulomb potentials. The more recently reported CoMSIA approach calculates similarity indices in the space surrounding each of the aligned molecules within the experimental set, and in addition to steric and electrostatic properties it calculates also hydrogen bond donor, hydrogen bond acceptor and hydrophobic fields [33]. CoMSIA is believed to be less affected by changes in molecular alignment and provides smooth and interpretable contour maps due to employing a Gaussian-type function. Using this approach we produced the first computer-aided and species-specific pharmacophore analysis of JH and its agonists. These revealed that the most active compounds for *Drosophila* need to have an electronegative atom (oxygen or nitrogen) at both ends of the molecule. When these electronegative atoms are replaced by carbon, or the distance between them is shorter than 11.5 Å or longer than 13.5 Å, their biological activity is decreased dramatically. They also showed that an electron deficient moiety in the middle of the JH agonist molecule is essential for high biological activity. The information obtained from CoMFA and CoMSIA contour maps identified the steric and electrostatic properties that are important features of ligand-binding cavity of JH target protein, a putative JH receptor. To refine this pharmacophore and evaluate alignment and the outcomes of the CoMFA and CoMSIA studies, we used the pseudoreceptor modeling software PrGen to generate a putative atomistic binding site model.

## Results

### Biological activity and structural diversity of JH agonists

**Figure 1:**
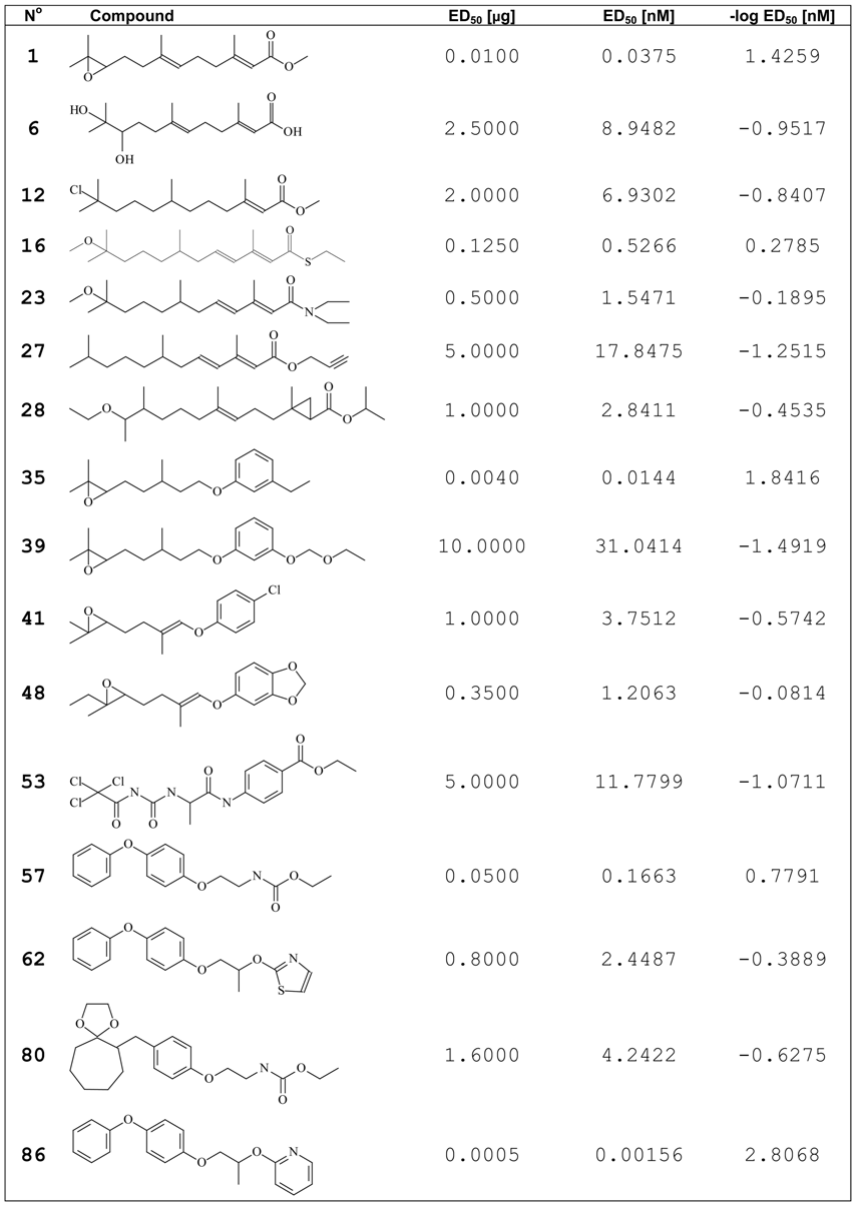
List of 16 representative JH agonists and their biological activities in *Drosophila* morphogenetic assay. The biological activity (ED_50_) is expressed in µg of the compound per animal, then it is converted to nmol per animal, and finally to -log of nmol values that are used for CoMFA and CoMSIA computations. Compounds 1–28 represent Class I agonists, whereas compounds 35–86 within this Table represent Class II agonists. Complete list of tested JH agonists is provided under Supporting Table S1. 1 = (*2E,6E*)-9-((*2R*)3,3-Dimethyl-oxiranyl)-3,7-dimethyl-nona-2,6-dienoic acid methyl ester (JH-III, also known as methylepoxyfarnesoate). 6 = (*2E,6E*)-(*S*)-10,11-Dihydroxy-3,7,11-trimethyl-dodeca-2,6-dienoic acid (JH-III acid diol). 12 = (*E*)-(*R*)-11-Chloro-3,7,11-trimethyl-dodec-2-enoic acid methyl ester. 16 = Tioethyl-(*2E,4E*)-(*R*)-11-methoxy-3,7,11-trimethyl-2,4-dodecadienoate (triprene; ZR-619). 23 = (*2E,4E*)-(*R*)-11-Methoxy-3,7,11-trimethyl-dodeca-2,4-dienoic acid diethylamide (ZR-618). 27 = (*2E,4E*)-(*R*)-3,7,11-Trimethyl-dodeca-2,4-dienoic acid prop-2-ynyl ester (kinoprene; ZR-777). 28 = 2-((*E*)-(*8R*,*9S*)-9-Ethoxy-4,8-dimethyl-dec-3-enyl)-2-methyl-(*2R*,*3S*)-cyclopropanecarboxylic acid isopropyl ester (ZR-4429). 35 = (*R*)-3-[5-(3-Ethyl-phenoxy)-3-methyl-pentyl]-2,2-dimethyl-oxirane. 39 = (*R*)-3-[5-(3-Ethoxymethoxy-phenoxy)-3-methyl-pentyl]-2,2-dimethyl-oxirane. 41 = (*R*)-3-{[(*E*)-4]-4-Chloro-phenoxy)}-3-methyl-but-3-enyl]-2,2-dimethyl oxirane. 48 = 5-[(*E*)-4-((*2R*,*3S*)-3-Ethyl-3-methyl-oxiranyl)-2-methyl-but-1-enyloxy]-benzo-[1], [3]-dioxole. 53 = (*S*)-4-{2-[3-(2,2,2-Trichloro-acetyl)-ureido]-propionylamino}-benzoic acid ethyl ester. 57 = [2-(4-Phenoxy-phenoxy)-ethyl]-carbamic acid ethyl ester (fenoxycarb). 62 = (*R*)-2-[1-Methyl-2-(4-phenoxy-phenoxy)-ethoxy]-thiazole. 80 = (*S*)-{2-[4-(1,4-Dioxa-spiro-[4], [6]-undec-6-ylmethyl)-phenoxy]-ethyl}-carbamic acid ethyl ester. 86 = 2-[1-Methyl-2-(4-phenoxy-phenoxy)-ethoxy]-pyridine (pyriproxyfen; Sumitomo 31183)

**Figure 2:**
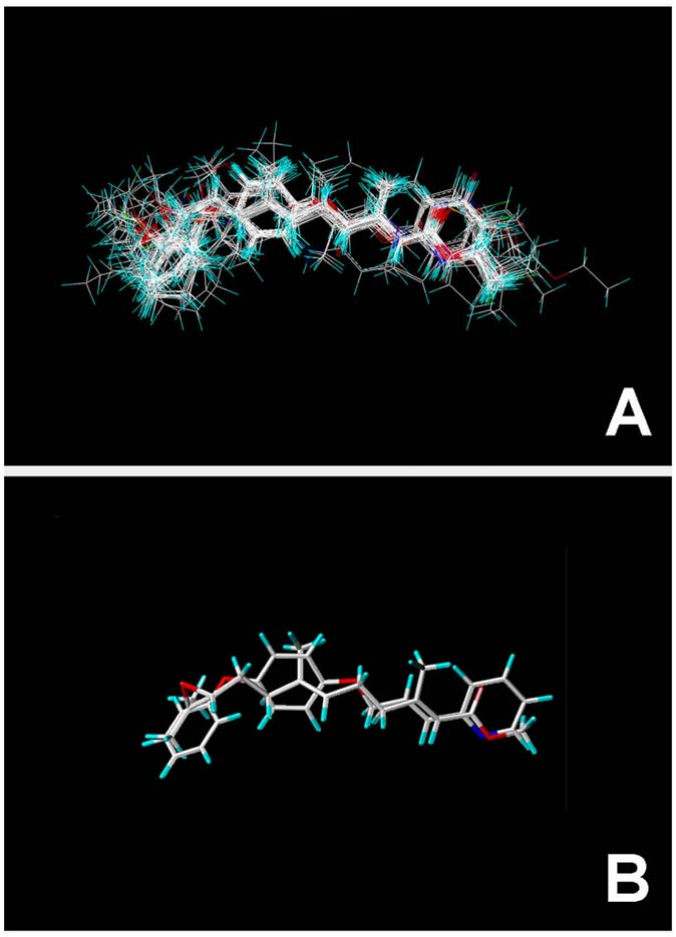
The superpositional alignment of congeners of JH agonists analyzed in this study. Complete set of all 86 JH compounds is shown in A, whereas B shows alignment of two selected agonists, natural JH-III (1) as representative of Class I and the most rigid structure of ZR-10852 (82) as representative of Class II.

We tested the biological activity of set of 86 JH analogs whose members spanned the range of structural diversity seen in JH agonists (Figure 1 and Figure 2A, see also Supporting Table S1). Although JH agonists belong to various chemical entities (farnesol and geraniol derivatives, trimethyl or tetramethyl-dodecenoate or undecenoate derivatives, juvabions, various derivatives of benzoic acid, acetophenone, aniline, nitrophenol, halophenol, benzenesulphonic acid or carbamate, then ω-alkoxy-ω,ω-dimethyl derivatives, oxime ethers, phenoxyphenoxy and other oligocyclic derivatives and peptidic juvenoids), from a structural point of view, the compounds we tested can be divided into two large classes. The first are linear flexible terpenoid or terpenoid-related molecules with several freely rotable bonds that include also natural JH-I, JH-II and JH-III (Class I structures **1–3**). The second are class with more rigid compounds containing phenoxy or other cyclic groups on both ends of the molecule exemplified by ZR-10183, ZR-10131 and pyriproxyfen (Class II structures **81**, **85**, **86**). Though many CoMFA reports have shown that it is difficult to use the analysis of flexible molecules to generate a CoMFA model, and many of the molecules used in this study have linear, flexible structure, the model presented here that is based on the superposition of both structurally diverse compound classes has acceptable predictive ability. Figure 1 shows the biological activity of selected 16 agonists whereas Supporting Table S1 shows the biological activity of all tested 86 agonists. This activity is expressed as ED_50_, and ranges from 0.00005 to 10 µg (0.0002 to 40.8163 nM) per animal where picogram amounts reflect most active compounds. JH agonists that show biological activity above 1 µg/animal can be considered as non-active.

### Optimizing the CoMFA model

We found that highly active compounds in both Class I and Class II have an electronegative oxygen at both of their ends or a nitrogen replacing the oxygen at one end (**1**–**3**, **14**–**17**, **19**, **81**–**86**). The biological activity dramatically decreases when these oxygens or the nitrogen are replaced by carbon (*e.g.* **25**, **26**, **29**, **31**, **32**, **43**, **44**). We were therefore interested in understanding how these atoms contributed to different longitudinal shifts of the structures in the alignments.

The nitrogen present in JH agonists is mostly part of an unsaturated heterocycle, carbamate or amide whereas the oxygen is mostly found within esteratic, etheric, epoxide or phenoxyphenol groups (see Figure 1 and Supporting Table S1). The oxygen in the phenoxyphenol group within Class II compounds is strongly sterically hindered by benzene rings and its free electron pairs participate in the conjugation system with phenyl rings. This makes the phenoxyphenol oxygen poorly reactive for intermolecular interactions including hydrogen bonding. On the other hand, the oxygen in the epoxy moiety in Class I compounds will provide electron pairs for H-bonding or for other electrostatic interaction very easily. On the opposite side of all compounds the oxygen is part of an esteratic group whereas the nitrogen is part of a carbamate, amide group or heterocycle where all these provide relatively weak interaction potential. Thus, the oxygen and nitrogen in the different compounds have very different chemical reactivities, atom charges and abilities to form hydrogen bonds or electrostatic interactions. These different properties pose challenges for generating an acceptable alignment of JH agonists. Since structural alignment is crucial step in CoMFA, we considered these aspects of the properties of JH agonists as we constructed multiple CoMFA models to identify an appropriate alignment.

Initially, we generated several variants of an alignment containing the entire set of compounds (Supporting Figure S1). We optimized a CoMFA model by creating a training set from 76 compounds and a test set containing ten structures representing both classes of compounds (linear and cyclic). All of the alignments were produced with respect to the electronegative oxygens or nitrogens located at the ends of the molecules. The alignment with the best statistical parameters was used for the final CoMFA model.

Many flexible linear compounds (*e.g.* **14**, **15**, **17**, **19**, **22**, **23**, **26**) are 1.5× longer in their extended conformation than typical rigid cyclic compounds (**56**, **66**, **72**, **74**, **80**–**86**). Thus, shorter bent conformations with low energy were selected as they fit better sterically to the cyclic compounds and improve *q^2^*. Varying the energy cut off from 10 to 30 kcal/mol did not have a significant effect on the predictive ability of the model. The best *q^2^* was achieved with a column filtering of 1 kcal/mol. A non-cross-validated PLS analysis was performed, and the final parameters and statistics (*q^2^* = 0.508, *r^2^* = 0.948) for the common training set (designated Class I+II) are summarized in the first row of Table 1. The predictive ability was externally evaluated through the prediction of a test set consisting of 10 ligands representing both Class I+II compounds with CoMFA predictive coefficient *r^2^* = 0.49 and CoMSIA predictive coefficient *r^2^* = 0.51 (Supporting Table S3) or of 5 Class I ligands with CoMFA *r^2^* = 0.54 and CoMSIA *r^2^* = 0.59 (Supporting Table S4), and 5 Class II ligands with CoMFA *r^2^* = 0.60 and CoMSIA *r^2^* = 0.63 (Supporting Table S5).

**Table 1:** Summary of results from CoMFA analyses for the common, unseparated training set of 76 JH agonists as well as for this set split into Class I and Class II compounds.

			Cross-validated		Non- cross-validated			Fraction	
Class	n	Comp	*q^2^*	SPRESS	*r^2^*	SEE	F test	S	E
**I+II**	76	6	0.508	0.969	0.948	0.306	196,93	0.426	0.574
**I**	45	6	0.576	1.007	0.983	0.202	325.47	0.403	0.597
**II**	31	6	0.686	0.767	0.987	0.155	308.77	0.524	0.466

Legend:

n = number of compounds.

Comp = number of PLS components in analysis.

*q^2^* = Squared correlation coefficient of a cross-validated analysis.

SPRESS = Standard deviation of error of prediction.

*r^2^* = Standard correlation coefficient of a non-cross-validated analysis.

SEE = Standard deviation of a non-cross-validated analysis (Standard error of estimate).

Fraction = Field contribution from CoMFA, S = steric, E = electrostatic.

In a second approach, we considered the above mentioned diversity of JH agonist structures and split their common alignment into two separated alignments. One covered each of the structurally diverse Class I and Class II compounds. This split led to separate CoMFA calculations and allowed us to explore how JH agonist structural diversity affected the CoMFA result. The two independent training sets showed nearly ideal alignment and markedly better statistical parameters than the initial, common training set. When CoMFA calculations of separated Class I and Class II subsets were performed, the final statistical parameters for each individual set (Class I: *q^2^* = 0.576, *r^2^* = 0.983; Class II: *q^2^* = 0.686, *r^2^* = 0.987) are significantly better than for entire set (I+II) (see rows two and three of Table 1). The calculated versus actual −log ID values of compounds in the test sets of Class I and Class II are shown in Supporting Figures S2 and S3, and the calculated versus actual −log ID values for all tested agonists are shown in Supporting Table S2.

### Optimization of the CoMSIA model

Standard steric and electrostatic fields, donor and acceptor hydrogen-bonding fields and hydrophobic fields were tested in the CoMSIA model and optimized as a function of energy cut off and column filtering. The statistical evaluation for the CoMSIA analyses was performed on the different versions of the dataset chosen during optimizing of the CoMFA model.

When we analyzed the whole set of structures, both Class I and II, the indicator fields yielded the most promising statistical results. Table 2 summarizes the optimized parameters and *q^2^* values for the indicator fields. A model with a *q^2^* value greater than 0.3 is usually considered to be significant, so models with field indicators that led to *q^2^*<0.3 were not considered. The best PLS analysis gave the following values at n = 76: *q^2^* = 0.534, *r^2^* = 0.901. Electrostatic factors played a major role as in the best CoMSIA model, the contribution of electrostatic field gave a high value of 0.740 *versus* 0.260 for steric factors (Table 2). Another significant descriptor was an acceptor hydrogen bond field although its *q^2^* was only 0.391. The graphical contour maps of the sterical and electrostatic fields are similar to the corresponding CoMFA plots. The significant *q^2^* value of a combination of steric, electrostatic and hydrogen bonding descriptors illustrate that these variables are necessary to describe interaction of JH compound with its target.

**Table 2:** Summary of results from CoMSIA analyses for a training set of 76 JH agonists.

			Cross-validated		Non- cross-validated			Fraction		
Class	n	comp	q^2^	SPRESS	*r^2^*	SEE	F test	S	E	DA
**I+II**										
S	76	2	0.371	1.065	0.744	0.805	31.07			
E	76	3	0.507	0.882	0.884	0.651	61.77			
SE	76	6	0.534	0.877	0.901	0.408	96.76	0.26	0.74	
A	76	3	0.391	1.072	0.667	0.725	45.42			
SEDA	76	3	0.485	0.901	0.736	0.643	63.86	0.08	0.36	0.56
**I**										
S	45	3	0.385	1.009	0.786	0.721	29.91			
E	45	6	0.551	0.934	0.926	0.396	70.80			
SE	45	6	0.637	0.875	0.960	0.290	136.18	0.29	0.71	
A	45	2	0.329	1.192	0.625	0.915	29.94			
SEDA	45	3	0.493	1.050	0.860	0.623	49.58	0.05	0.23	0.72
**II**										
S	31	6	0.755	0.678	0.956	0.286	87.37			
E	31	2	0.675	0.722	0.841	0.505	74.23			
SE	31	2	0.695	0.700	0.927	0.396	78.38	0.20	0.80	
A	31	3	0.537	0.878	0.836	0.522	45.91			
H	31	6	0.660	0.798	0.978	0.205	174.16			
DA	31	3	0.481	0.930	0.843	0.511	48.28			
SEDAH	31	3	0.579	0.837	0.887	0.435	70.31	0.07	0.25	0.51

Legend:

n = number of compounds.

Comp = number of PLS components in analysis.

*q^2^* = Squared correlation coefficient of a cross-validated analysis.

SPRESS = Standard deviation of error of prediction.

*r^2^* = Standard correlation coefficient of a non-cross-validated analysis.

SEE = Standard deviation of a non-cross-validated analysis.

Fraction = Field contribution from CoMSIA like S = steric, E = electrostatic, A = hydrogen bond acceptor type, D = hydrogen bond donor type, H = hydrophobic.

When the set of agonists was divided into Class I and Class II structures and separate CoMSIA calculations performed, the differences between these two classes became more remarkable. This can be seen in the different types of indices producing the best models of separated agonist classes. The final statistical parameters of both classes (Class I: n = 45, *q^2^* = 0.637, *r^2^* = 0.960 for steric and electrostatic indices; Class II: n = 31, *q^2^* = 0.755, *r^2^* = 0.956 for steric index) are summarized in Table 2 and graphically interpreted in Supporting Figures S4 and S5.

### 3D QSAR outcomes

**Figure 3:**
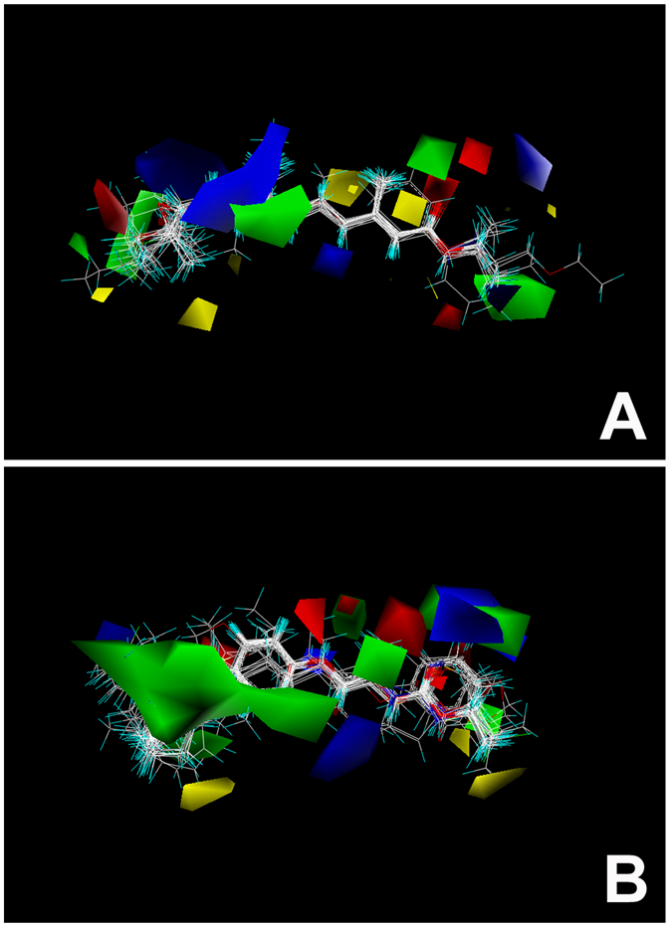
CoMFA steric and electrostatic fields contour plot for Class I and Class II JH agonists. Green polyhedra represent sterically favored regions where more bulky substituents increase biological activity, while yellow polyhedra surrounded regions indicate sites where less bulky substituents are appreciated for increasing biological activity. Blue polyhedra represent electrostatic regions where positively charged groups will be favorable and will enhance biological activity, whereas the red contours represent regions where negative charge is favorable. The importance of the electronegative oxygens or nitrogens on the ends of the aligned structures is indicated by red polyhedra near the positions of these atoms. The presence of smaller red polyhedra in the middle of the JH agonist structure indicates an additional site where an electronegative atom or group can enhance biological activity. The contour map of Class II (B) differs from that of Class I (A) mainly in the steric fields. The large green polyhedra of Class II (B) near the part of the phenoxyphenyl group (left side) indicates that the presence of steric bulk substituents in this part of the molecule enhances biological activity.

In order to visualize the information content of derived CoMFA model, 3D electrostatic and steric contour maps were generated (Figures 2A and 2B). The importance of the electronegative oxygens or nitrogens at the ends of the aligned structures is indicated by red polyhedra near the positions of these atoms. The presence of smaller red polyhedra in the middle of a JH agonist structure indicates an additional site where an electronegative atom or group enhances the biological activity, as exemplified by the oxygen in the middle of SJ-68 oxid (**9**) or double bond in JH-III (**1**). The contour map of Class II (Figure 3B) differs from that of Class I (Figure 3A) mainly in the steric fields. The large green polyhedra seen in the Class II map lie near the phenoxyphenyl group and indicate that the presence of steric bulk substituents in this part of the molecule enhance biological activity.

CoMSIA contour maps are easier to interpret than CoMFA maps as they partition variance into the different field types. The contour maps for the steric and electrostatic CoMSIA fields are shown in Figures 3A (Class I) and 3B (Class II). The green polyhedra, as in the CoMFA contour maps, represent sterically favored regions in which more bulky substituent increase biological activity, while yellow polyhedra represent sterically disfavored regions where a less bulky substituent can increase activity. In the CoMSIA electrostatic contour plot, red polyhedra represent favorable regions where negatively charged groups enhance activity while blue polyhedra represent disfavored regions where positively charged groups enhance activity. The steric and electrostatic fields of CoMSIA maps are generally in good accordance with the field distribution of CoMFA maps. They do, however, indicate more sterical freedom for Class II compounds on their phenoxyphenol side.

**Figure 4:**
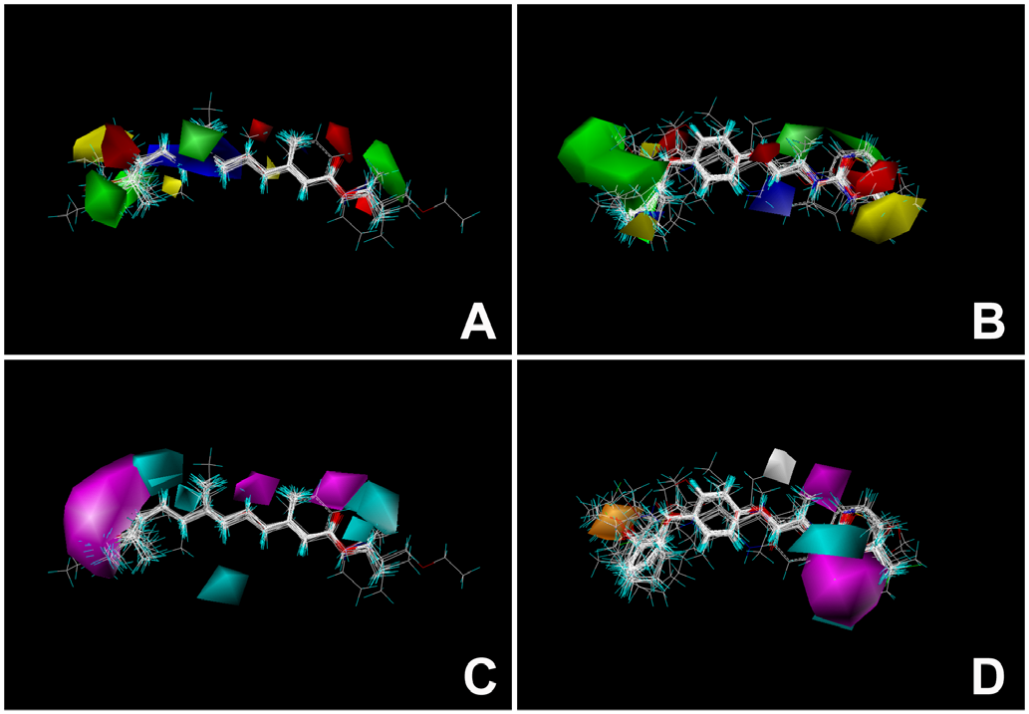
Contour maps of Class I and Class II JH agonists CoMSIA analyses. Green polyhedra represent sterically favored regions in which more bulky substituents will increase biological activity, while yellow polyhedra represent sterically disfavored regions where less bulky substituents are appreciated for increasing the activity of both Class I (A) and Class II (B) JH agonists, respectively. In the electrostatic contour plot, the red polyhedra represent favorable regions where negatively charged groups will enhance activity and the blue polyhedra represent disfavored regions where positively charged groups will enhance activity. Contour maps for the hydrogen bond acceptor and donor fields are illustrated in C (for Class I agonists) and D (for Class II agonists). Magenta areas indicate regions where hydrogen bond acceptors are favorable for increasing biological activity (oxygens and nitrogens in the ligand), cyan areas indicate fields where hydrogen bond donors are favorable (NH and OH groups in ligand). Orange polyhedra surround area where H-bond acceptors are unfavorable and white polyhedra areas where H-bond donors are unfavorable.

**Figure 5:**
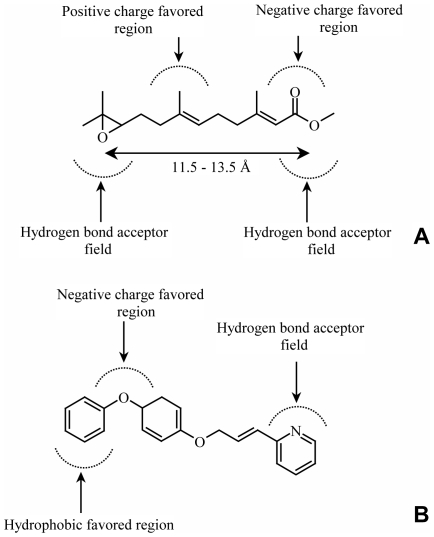
Pharmacophore models of Class I and Class II JH agonists based on CoMFA and CoMSIA result. Models show key structural elements responsible for the hormonal activity of JH agonists. Both classes of JH compounds share regions that favor negative charge and a field that requires hydrogen bond acceptor. In both cases these elements are located at the sides of JH agonists. However, Class I compounds (A) have also additional hydrogen bond acceptor element located on the esteratic side, and a region which favors positive charges centrally. A specific feature of Class II compounds (B) is a hydrophobic region that is favored around the outside edge of a phenoxyphenol moiety. Due to the high flexibility of Class I compounds the distance between hydrogen bond acceptor atoms (*e.g.* esteratic and epoxy oxygens at two sides) must be between 11.5 and 13.5 Å to possess agonist biological activity in *Drosophila*. Highly rigid Class II compounds that show JH biological activity in *Drosophila* automatically fit to this requirement.

A more dramatic difference between Class I and Class II compounds appears in the CoMSIA hydrogen bond acceptor and donor fields (Figures 3C and 3D). These highlight the areas beyond ligands where putative hydrogen bond partners (amino acid residues) in the putative receptor could form hydrogen bonds. Magenta areas indicate where hydrogen bond acceptors are favorable for increasing biological activity (oxygens and nitrogens in ligand), while cyan areas indicate fields where hydrogen bond donors are favorable (NH and OH groups in the ligand). Orange polyhedra surround the area where H-bond acceptor is unfavorable and white polyhedra the area where H-bond donor is unfavorable. In the Class I contour map (Figure 4C) the importance of the hydrogen bond acceptor interaction can be seen at both ends of the molecules, in the positions of the oxygen within the esteric group and in the position of the epoxy or etheric oxygen on the other side. This is also seen when the ED_50_ values of compounds **15** and **22** are compared. The only difference between these two compounds is that an esteric oxygen (a hydrogen bond acceptor) is replaced by an amidic nitrogen (a hydrogen bond donor), which leads to 100-fold decrease in the biological activity of **22**. This is also apparent from both of the CoMFA and CoMSIA electrostatic contour maps in which a negative charge enhances activity (red color). When these electronegative atoms in Class I structures are replaced by carbon (*e.g.* compounds **21**, **25**, **26**, **29**, **31**, **32**) or distance between these electronegative end points was shorter than 11.5 Å or longer than 13.5 Å (compounds **28**, **40**, **41**, **48**, **49**), biological activity dropped down dramatically. In addition, the presence of an electronegative oxygen or a double bond in the middle of the structure (*e.g.* **9**, **15**, **17**) which results in a site with an increased concentration of negative charge, and less electron charge at the ends, also seems to be important for biological activity. These areas are represented by blue polyhedra on the CoMFA and CoMSIA contour maps. The contour map of Class II lacks polyhedra for a favorable H-bond donor or acceptor field on phenoxyphenol side of structures. The CoMFA and CoMSIA calculations indicated that the electrostatic requirements are more important than the steric ones because of the electrostatic fraction had higher values than the steric fraction for both Class I and Class II (Tables 1 and 2). The CoMFA electrostatic fraction is 0.574 versus a steric fraction of 0.426. This is even more remarkable in the CoMSIA calculation where the electrostatic fraction is 0.740 while the steric fraction is 0.260. Thus, the crucial element that contributes to high affinity binding is the presence of negative charge-rich atoms or groups at the ends of the molecules represented by electronegative atoms of oxygen, nitrogen, or unsaturated cycle. As this is also supported from CoMSIA field indices and hydrogen bond acceptor fractions (Table 2), these electronegative atoms are most probably involved in hydrogen bonding with the putative receptor. Thus, the analysis of CoMFA and CoMSIA models for Class I+II revealed the points and regions that are highly correlated to the activity of tested compounds. These data from 3D QSAR analyses suggested two pharmacophore models that depict the key structural requirements for the biological activity of JH agonists (Figure 5A and 5B).

### Pseudoreceptor model

We then used the pharmacophore model with the PrGen program to produce an atomistic pseudoreceptor model. This model contains the essential features of an active site by assuming complementarity between the shape and properties of the receptor site and the bioactive conformations of the set of JH compounds, and thus mimics the real receptor binding surface. The alignments of natural JH-III, the five most active ligands of Class I, and the six most active ligands of Class II were used to generate corresponding pseudoreceptors. We selected the appropriate amino acid residues for pseudoreceptor construction by considering two important factors. First, we used data derived from the CoMFA and CoMSIA structure-activity relationships. Second, we used information about which amino acids are most frequently involved in forming interactions between members of the nuclear receptor superfamily and small lipophilic ligands. Based on these criteria, polar amino acids (*e.g.* Arg, Asp) were placed complementarily to the vectors of atoms with local electron deficit, and residues acting as H-bond donors (*e.g.* Tyr, Asn) were placed complementarily to the vectors of esteratic or etheric oxygens. Hydrophobic amino acids (*e.g.* Met, Leu, Ile, Tyr) were gradually located around the rest of the molecular alignment. For acceptor type of hydrogen bond interactions, we used bonding with Ser, Thr, Tyr, His, Arg or Gln, and we used Ser, Thr, Tyr, His, Arg, Gln, Lys or Asp to mediate electrostatic interactions. After the whole complex of a pharmacophore surrounded by an active site was generated, the energy equilibration protocol was applied to produce an energetically relaxed model until the best possible correlation between calculated and experimental free energy of ligand binding could be achieved. A variety of physical phenomena are thought to contribute to the binding affinity (*K_B_*) of an interaction, including those that are considered to make a largely enthalpic contribution, for example, van derWaals interactions, hydrogen bonding and electrostatic complementarity, and those considered to be dominated by entropy, for example, changes in configurational disorder and in the solvation of hydrophobic/lipophilic groups upon formation of the complex [34], [35]. The binding affinity, or equilibrium binding constant (*K_B_*), describes the ratio of concentration of a complex (PL) at equilibrium for a reversible reaction between free protein (P) and ligand (L) *K_B_* = [PL]/[P] [L]. At equilibrium under conditions of constant pressure, the binding constant *K_B_* is related to the standard Gibbs free-energy change (*ΔG^o^*) of the reaction through *ΔG^o^* = −*RT*ln*K_B_* where *R* is the gas constant (8.314472 J mol^−1^ K^−1^) and T is the temperature (in Kelvin) [36]. The conversion of the dissociation constant *K_d_* to free- energy of binding is apparent from equation *ΔG^o^* = *RT*ln*K_d_* [37]. The more negative the value of *ΔG^o^*, the more favorable the reaction. The change in free energy itself is composed of enthalpic (*ΔH^o^*, effectively, the heat given out or taken up upon making and breaking bonds) and entropic (*ΔS^o^*, which represents the energetic consequences of changes to the degree of order within the system) changes, where *ΔG^o^* = *ΔH^o^*−T*ΔS^o^* [34], [38]. To find the optimal energy equilibrium, this procedure was repeated iteratively with each amino acid combination selected for their appropriate positions at the tips of vectors.

**Figure 6:**
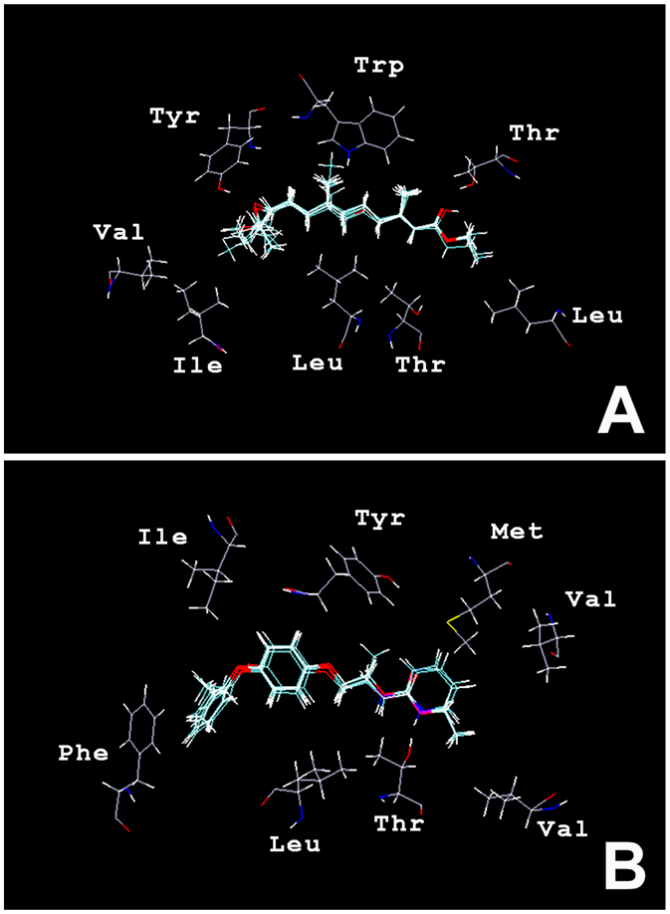
Three-dimensional structure of the pseudoreceptor models for Class I (A) and Class II (B) JH agonists. Selected compounds are aligned in the middle with the surrogate of eight amino acids surrounding them. Both models are composed of eight amino acid residues reflecting all interactions that are predicted to be required from pharmacophore analysis. For the sake of clarity, bonding interactions and vectors have not been displayed.

A combination of Trp, Thr, Leu, Thr, Leu, Ile, Val and Tyr amino acid residues yielded the best correlation coefficient (*r^2^* = 0.91) and had a predictive cross-validated coefficient of *q^2^* = 0.53 between the experimental free energies of binding and predicted free energies of binding for an atomistic pseudoreceptor model of Class I ligands (Figure 6A). In this model, the hydrogen bond donors Tyr and Thr are positioned in the vicinity of the esteratic or amidic group of the aligned molecules, Tyr is positioned near the epoxy group on the opposite site of the molecular alignment. Trp is positioned optimally near the electron deficient middle part of the Class I alignment. When the same procedure was applied to data from the Class II pharmacophore model, a combination of Tyr, Met, Val, Val, Thr, Leu, Phe and Ile amino acid residues yielded the best statistical correlation coefficients *r^2^* = 0.92 and cross-validated predictive coefficient *q^2^* = 0.43. As in the pseudoreceptor model based on the Class I structures, in the model based on Class II structures (Figure 6B) Thr is placed as a hydrogen-bond donor in the vicinity of the esteratic or amidic group of the agonist alignment, and Tyr is placed near the middle electron deficient region. The hydrophobic residues (Ile, Phe, Leu) are positioned against the phenoxyphenyl group. The theoretical relative Gibbs free energies calculated for the binding of both series of agonists *vs* experimental relative Gibbs energies are shown in Tables 3 and 4.

**Table 3:** ΔG Experimental versus ΔG PrGen predicted activities for JH agonists of Class I.

Comp. No	ΔG_exp_	ΔG_pred_
**1**	2.02	1.44
**2**	−1.09	−1.42
**8**	−2.17	−2.72
**9**	−5.25	−4.70
**15**	3.40	3.86
**19**	2.54	1.73

**Table 4:** ΔG Experimental versus ΔG PrGen predicted activities for JH agonists of Class II.

Comp. No	ΔG_exp_	ΔG_pred_
**53**	−2.05	−2.08
**54**	−1.90	−2.16
**56**	−1.50	−1.80
**82**	−2.41	−1.98
**84**	−2.26	−1.08
**86**	−3.98	−3.94

## Discussion

This study sought to establish an initial juvenoid 3D QSAR by CoMFA and CoMSIA models of extensive molecule set. Early trials to look at action of JH agonists via structure-activity relationships confronted several issues [19], [39]–[41]: (i) The largest obstacle was in testing, collecting and comparing data simultaneously among several species. Especially at the early stages of an analysis of structure-activity relationships, this approach hampered the ability of obtaining interpretable data specific for a given insect species. Since the molecular mode of JH action relies on an interaction between a small ligand and its protein target, there are likely species-specific differences in amino acid sequence and protein structure that makes it extremely difficult to develop a pharmacophore model useful for predicting the molecular properties of a JH receptor. (ii) A second and often neglected problem was involved in selecting the characteristic used to measure ID_50_, IC_50_ or ED_50_. For simplicity and efficient data collection, researchers were frequently satisfied with counting the inhibition of metamorphosis or the prevention of eclosion. These easily could reflect a compound's insecticidal toxicity rather than its morphogenetic action, generating misleading data. (iii) A third problem is associated with method of juvenoid application. This can make a crucial difference in testing JH biological activity. For example, very different dose-response curves and ID_50_ or IC_50_ values will be obtained when animals are given a tested compounds via a single direct topical application than via its continuous presence in food or the living environment (*e.g.* in the water for aquatic insects [42]). Under conditions where a compound is continuously present, there is an increased chance that toxic effects will have prevalence over realistic biological activity. (iv) Until now, structure-activity studies focused on testing JH analogs in agricultural pests or disease vectors that, in contrast to *Drosophila*, could not provide suitable genetic or molecular tools to dissect the molecular mode of JH action. There is good chance that combination of present approach with the power of *Drosophila* genetics can be explored in near future. (v) Finally, versatile bioinformatics-aided computer programs to evaluate data were unavailable when there was highly active research on developing JH analogs. To avoid these pitfalls, we chose to use just one insect species, and chose *Drosophila* since it is a well characterized genetic model. To be certain we were evaluating real hormonal activity of compounds, we used a strictly defined, specific morphogenetic effect [23], [25], [27], [43] as a criterion for our ED_50_ calculations. Finally, to avoid any misinterpretation, we generated, collected, processed and evaluated all data in using a well-established 3D QSAR computational approach exclusively within our research group.

In many recent CoMFA and CoMSIA studies, researchers have analyzed data sets from *in vitro* assays where a recombinant receptor is used and transiently expressed in host cells and where a series of compounds is tested for reporter activity. While this approach makes predictions much easier and straightforward to test, and is widely used in drug development, lipophilic compounds pose a unique challenge. Here, we employed a reverse approach where quantitative structure-activity data and results from CoMFA/CoMSIA analyses shed light on the properties of a putative JH receptor.

For a long time, it has been thought that the biological activity of JH analogs in *in vivo* tests is strongly affected by their solubility, penetrance through cuticle, transport via hemolymph and delivery to target tissues (for reviews see [18]–[20], [22], [44]). In fact, many of these conclusions were based on the lipophilic properties of JH compounds and analogs. Notwithstanding this potential complication, the CoMFA and CoMSIA analyses presented here demonstrate the hydrophobic and donor-acceptor interactions of Class I JH agonists have very poor *q^2^* and *r^2^* values. It is already known that these interactions, under certain circumstances, may reflect non-specific interactions of biological molecules such as solubility, penetrance, and transport *etc*. [45]–[50]. In contrast, the highly significant *q^2^* and *r^2^* values for the steric and electrostatic fields document that the QSAR data obtained reflect the real binding of a ligand to its protein target, possibly a receptor, rather than non-specific interactions [51]. Two important considerations shed light on this result. First, JH compounds that show biological activity are active at relatively low concentrations, concentrations that are below the threshold that would produce additive or non-specific effects. This is consistent with the negligible role that hydrophobic interactions are observed to play in biological activities. Secondly, the composition of the *Drosophila* exoskeleton (its cuticular layers) [52], [53] appears to be relatively favorable for JH penetrance and offers little if any resistance to hormonal effects. This notion is supported by the observation that the application of JH compounds topically or in drinking water renders a very different biological activity response in different insects (*e.g.* to the linden bug, *Pyrrhocoris apterus* [18], [20]). Therefore, 3D QSAR analysis of *in vivo* biological activities of juvenoids in insects from other systematic groups may not be as effective in developing CoMFA or CoMSIA models.

One of the most fascinating questions in research on JH agonists has been to understand what these structurally divergent compounds have in common that enables them to retain identical biological activity? Our pharmacophore model based on SAR of the most active Class I agonists has 5 elements. These 5 elements are: (i) acceptor type of hydrogen bond related to esteratic or equivalent oxygen (as shown from studies with **17**, **23** and **24**); (ii) a second acceptor hydrogen bond originating from the epoxy oxygen on the opposite side of the molecule (as apparent in **35**); (iii) an electrostatic interaction from a carbonyl (keto) oxygen on the same side of the molecule (see **1**–**12**, **15**, **17**, **20**, **22**, **25**, **26**) which potentially may become hydrogen bonding element; (iv) a strict distance (11.5–13.5 Å) between these hydrogen acceptor oxygens or nitrogens; (v) positively charged groups are favored in the middle of the molecule. JH-III as a natural ligand has only 4 of these elements which explains why several JH agonists, in addition to commonly known resistance to metabolism [12], [18], [20], can be more potent than natural JH. Our pharmacophore model of Class II agonists has 3 elements: (i) as for Class I agonists, the first element is a hydrogen bond acceptor on the right side of the ligand; (ii) a negatively charged group on the opposite side of the pharmacophore; and (iii) a bulky hydrophobic region on the same side where the phenoxyphenol moiety is usually found.

Pseudoreceptor modeling [54]–[56] is one of several receptor mapping approaches, where a paucity of information concerning receptor structures has spawned techniques that project the properties of the bioactive ligands into three dimensions around their appropriately superimposed molecular framework. The molecular nature of natural JH and its agonists (similar to retinoids, free fatty acids and steroids) and the type of interactions predicted from pharmacophore models strongly argue that the ligand binding pocket of the receptor for JH holds properties that are similar to members of nuclear receptor superfamily. Consistent with this, we constructed a pseudoreceptor ligand-binding site by using information about the amino acids most frequently involved in forming interactions with small lipophilic ligands in nuclear receptors [57]–[60]. To achieve the optimal positions of the selected residues, a receptor equilibration was subsequently performed allowing for translation, rotation, and torsional variations of receptor's amino acid residues. The resulting map provides steric, electrostatic, and lipophilic profiles used to identify the type and approximate position of receptor residues, or their functional groups, interacting with the ligand. This map can be used for subsequent molecular modeling and allows for semiquantitative predictions of binding affinities for ligands. The structure of the resultant complex contains 8 amino acid residues that span the alignment of Class I as well as Class II JH agonists. Both models of a three-dimensional receptor surrogate have been validated, leading to high correlation and predictive power as well as a perfect agreement with the pharmacophore models. Therefore, the data that are presented here have allowed for the first time the rationalization of JH-agonist SAR not only at a qualitative level but also provided quantitative relationships between the structure of JH compounds and their biological activity as summarized and reflected in likelihood models of the docking pocket of a putative JH receptor. We believe that this approach, in combination with 3D-object recognition based on scanning, pairwise comparison or similar protocols [61], [62] to identify the spatial coordinates of individual atoms can be used in the near future to find potential siblings in structural databases.

## Materials and Methods

All experiments have been performed on the wild-type strain *Oregon R* of *Drosophila melanogaster*. JH and JH agonists (for complete list see Supporting Table S1), dissolved in acetone were applied topically in 0.5 µl onto the abdominal surface of late wandering 3rd instar larvae. Details on testing and evaluation, including the processing of animals for light and electron microscopy are provided in the Supporting Experimental Procedures S1 and References S1.

CoMFA and CoMSIA computations were performed with the molecular modeling software package Sybyl ver 6.8 (Tripos Co., St. Louis, MO, USA) on a Silicon Graphics Origin 2000 (R10000) and O_2_ (R10000) servers. The AM1 semiempirical method was applied for geometrical optimization and calculation of the partial atomic charges.

### Molecular alignment

Structural alignment is the most critical step in CoMFA study and the resulting model is often sensitive to a particular alignment. While it is recognized that the global energy-minimum conformation may not necessarily be adopted in the drug-receptor complex, the use of a reasonably low energy conformation in the alignment is a useful starting point for statistical comparisons of flexible structures within both the CoMFA and the CoMSIA models. In this study, we took lowest energy conformation of the most rigid and highly active molecule (No. **82**) as the template structure for the alignment. Molecules were superimposed by minimizing the root mean square (RMS) distance between atom pairs that belongs to the fitting molecule and to template molecule, respectively, and the alignment for all 86 compounds within the test set is shown in Figure 2A. Superimposition of ligands was based on manually selected overlapping oxygens or nitrogen at the ends and quaternary or *sp^2^* hybridized carbon in the middle of the structures (Figure 2B).

### CoMFA analysis

The CoMFA analysis of this set of molecules was carried out on the steric and electrostatic fields using the standard options of Sybyl 6.8 package. A three dimensional cubic lattice with a 2 Å grid spacing was generated automatically around aligned molecules with the grid extending molecular dimensions up to 4 Å in all directions. The steric and electrostatic fields were calculated separately for each molecule using *sp^3^* hybridized carbon atom with charge of +1. Energy cut off value of 30 kcal/mol was applied, which means the calculated energies greater than 30 kcal/mol are truncated to this value and thus avoiding infinity of energy values inside molecule. Partial least squares (PLS) method was used to analyze the relationship between the calculated steric and electrostatic energies and −log ED values. The crossvalidation PLS calculation was performed by a Leave-One-Out (LOO) procedure. To speed up the analysis and reduce noise, column filtering was set at 1.0 kcal/mol so that only those steric and electrostatic energies with values greater than 1.0 kcal/mol are considered in the PLS analysis. To maintain the optimum number of PLS components and minimize the tendency to overfit the data, the number of components corresponding to the lowest Predictive Error of Estimate (PRESS) value was used for deriving the final PLS regression models. Cross-validation determines the optimum number of components, corresponding to the smallest error of prediction and the highest cross-validated *q^2^*. The analyses were carried out with a maximum of ten components, and subsequently, using the optimal number of components at which the difference in the *q^2^* value to the next one was less than 5% and error of prediction was the lowest one. The models were estimated on the crossvalidated LOO *r^2^* (expressed as *q^2^*), standard error of prediction, SEP, the non-crossvalidated conventional correlation coefficient *r^2^*, and the standard error of estimate SEE.

The overall predictive ability of the analysis was evaluated by the term *q^2^* which was calculated according to following equation:
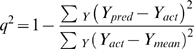
Whenever crossvalidation is used in conjunction with PLS, some above indices change and others are omitted as meaningless. The key difference is in the definition of standard error *s* value. In analysis without crossvalidation the standard error is uncertainty remaining after least squares fit has performed. In crossvalidation, standard error becomes the expected uncertainty in prediction for the individual compound based on the data available from other compounds. In this context *s* becomes the root mean Predictive Error of Estimate (PRESS). It is harder to predict values which are not used in deriving a model than it is to fit the same values which including the minimum model, and the crossvalidated correlation coefficient *q^2^* is always much lower than the conventional correlation coefficient *r^2^*. Uncertainty of prediction is defined as:
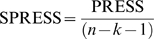
However, PRESS and *q^2^* are generally proving to be better indicators than standard error and *r^2^* of how reliable predictions actually are. A model with a *q^2^* value greater than 0.3 is usually considered to be significant. The CoMFA steric and electrostatic fields of the analysis are present as contour maps (Figure 3). The color polyhedra surround those lattice points where the QSAR strongly associates changes in compound field values with changes in biological potency. In the maps, color polyhedra surround the lattice points in which QSAR identifies fields where compounds have significant changes in biological activity. Green polyhedra represent sterically favored regions where more bulky substituents increase biological activity, while yellow polyhedra surround regions where less bulky substituents are able to increasing biological activity. Blue polyhedra represent electrostatic regions where positively charged groups are favorable and enhance biological activity, whereas the red contours represent regions where negatively charged groups are favorable.

### CoMSIA analysis

Analogous to CoMFA, a data table is constructed from similarity indices calculated at the intersections of a regularly spaced lattice 2 Å grid in CoMSIA. Unlike CoMFA, CoMSIA uses the Gaussian function for the distance dependence between the probe atom and the molecule atoms to avoid some of the inherent deficiencies arising from the functional form of the Lennard-Jones and Coulomb potentials. We take into evaluation 5 physicochemical properties: steric (S), electrostatic (E), hydrophobic (H), hydrogen-bond donor (D) and acceptor (A) properties. Using all five CoMSIA descriptors for the explanatory variables, a LOO run and a novalidation-PLS analysis were performed. These five different fields help to increase the model's significance and predictive power as well as to partition the various properties into the spatial locations where they play a decisive role in determining biological activity. Similarity indices A_F,K_ between the compounds and a probe atom are calculated according equation:
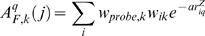
where *q* is the grid point for molecule j, with the summation index over all atoms of the molecule j; *w_ik_* the actual value of physicochemical property k of atom i; w_probe,k_ indicates probe atom with charge +1, radius 1 Å, hydrophobicity +1; H-bond donor and acceptor property +1; α is the attenuation factor, default is 0.3; *r_iq_* is the mutual distance between probe atom at grid point *q* and atom *i* of the test molecule [33]. Using all five CoMSIA descriptors for the explanatory variables, a LOO run and a novalidation-PLS analysis were performed. The models were estimated on the crossvalidated LOO *q^2^*, standard error of prediction (SPRESS), the non-crossvalidated conventional correlation coefficient *r^2^*, and the standard error of estimate (SEE). The color polyhedra surround those lattice points where the QSAR strongly associates changes in compound field values with changes in biological potency.

### Pharmacophore and pseudoreceptor model generation

Using the results of CoMFA and CoMSIA, we have proposed a putative pharmacophore model that explains the key structural requirements for the activity of JH and its agonists. To refine this pharmacophore models the program PrGen (3R Biographics Laboratory Foundation, Basel, Switzerland) was used to generate the pseudoreceptor. Based solely on the structures of ligand molecules this method aims to predict the relative free energies of binding. The model is validated by its ability to reproduce the experimental data of the set of ligands. The 3D coordinates of JH-III and five most active JH agonists from Class I and six most active JH agonists from Class II superimposed in the conformations from the CoMFA study were used in the alignment for PrGen pseudoreceptor modeling. From the overlapped ligands, the program generates vectors for each functional group indicating steric, electrostatic, and lipophilic interactions. Pseudoreceptor is then created from individually chosen residues that are positioned at the tips of the vectors. Residues were chosen specifically to fit the type of interaction of each vector as characterized by overlapped molecules and CoMFA based pharmacophore. The experimental free energies of ligand binding were calculated according to methodology reported by Vedani *et al*. [55], Bassoli *et al*. [63] and Zbinden *et al*. [64]. Experimental free energies of binding were calculated from the equation:
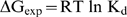
where K_d_ is experimental dissociation constant. The dissociation constants of JH agonists are unknown, for this reason dissociation constant K_d_ was approximated by lnED_50_.

In PrGen, free energies of binding, ΔG*_0_*, are estimated from equation:
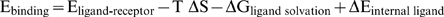
Algorithms to calculate these quantities are included in PrGen. Predicted free energies of ligand binding, ΔG_pred_, are obtained by means of a linear regression between ΔG_exp_ and E_binding_:
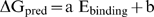
The resulting complex of superimposed ligand molecules and amino acids residues of the pseudoreceptor was optimized by a conformational search protocol combined with energy minimization. This step is repeated until the functional groups interact with a pseudoreceptor residue. An interactive algorithm, equilibration protocol, is used to obtain the best correlation between experimental and predicted free energies of binding.

## Acknowledgments

Authors would like to thank Dr. Karel Sláma of the Entomological Institute of Czech Academy of Sciences (Prague, Czech Republic) for generous supplying many JH analogs from his collection, and for continuous support during this work. We are also indebted to Dr. Clive A. Henrick of Sandoz Agro Inc. (former Zoecon Corporation, Palo Alto, CA, USA), Dr. William Bowers (Department of Entomology, University of Arizona, Tucson, AZ, USA), Dr. Hans Laufer (Department of Molecular & Cell Biology, University of Connecticut, Storrs, CT, USA) and Drs. Zdeněk Wimmer and Martin Rejzek (Institute of Organic Chemistry and Biochemistry, Czech Academy of Sciences, Prague, Czech Republic) for providing two more than dozens of JH compounds. Thanks go also to Dr. Thomas Flatt (Brown University, Providence, RI, USA) and Dr. Bruce A. Chase (University of Nebraska, Omaha, USA) for critical reading of the manuscripts and continuous support. Technical assistance of Erika Skácelová is greatly acknowledged.

## Supporting Information

Figure S1 Comparative plot of experimental versus CoMFA predicted biological activities (−log ED50) of common training set (Class I+II) of 76 JH agonists. Despite being structurally diverse, most active compounds in Drosophila share some common features, i.e. an electronegative atom (oxygen or nitrogen) at one end of the molecule and electronegative atom (epoxy oxygen) or electron rich moiety (oxyphenyl group) on the molecule's opposite end (see compounds 1–3, 14–17, 19, 81–86). Nonetheless, the terpenoid and rigid phenoxy structures have very different chemical reactivity, atom charges and abilities in forming hydrogen bonds or electrostatic interactions. Indeed, this was one major reason to divide the complete training set into two classes. The oxygen in phenoxyphenol group of Class II compounds is sterically hindered by benzene rings that makes the phenoxyphenol oxygen poorly reactive for intermolecular hydrogen bonding, while the oxygen within an epoxy moiety of Class I compounds can easily provide electron pairs for H-bonding or for other electrostatic interactions. The difference between Class I and II analogs is reflected also in their negative charge distribution. In the Class I structures it is concentrated near electronegative, ether or epoxy oxygen whereas in Class II structures it is localized to the phenyl rings. Indeed, a similar protocol for subdividing compounds into two chemotypes for QSAR analyses was published recently for COX-2 inhibitors [41] and steroid hormones to reflect the unusual conformational adaptation of nuclear receptor ligand binding domains to agonist variety [42], [43]. Furthermore, the presence of an electron deficient moiety in the middle of the JH agonist molecule is essential for the very high biological activity seen in some synthetic JH agonists but not observed in natural JH (blue and cyan polyhedra regions in CoMFA and CoMSIA contour maps, respectively; see Figures 3 and 4A, B). On the other hand, the steric CoMFA and CoMSIA contour maps indicate that presence of more bulky substituent in Class I compounds (green polyhedra) will enhance their biological activity. More bulky substituent (yellow polyhedra) near the phenoxyphenyl or epoxy groups in Class II compounds would decrease their biological activity (as again shown in Figures 3B and 4B). Thus, it is significant that each of these two independent training sets have shown a nearly ideal alignment and markedly better statistical parameters than the original common set.
(2.99 MB TIF)

Figure S2 Graphical representation of observed versus CoMFA predicted biological activities (−log ED_50_) for training set of Class I JH agonists.
(2.99 MB TIF)

Figure S3 Correlation between experimental and CoMFA predicted biological activities (−log ED_50_) for training set of Class II JH agonists.
(2.99 MB TIF)

Figure S4 Graphical representation of observed versus CoMSIA predicted biological activities (−log ED50) for training set of Class I JH agonists. The difference between Class I and Class II agonists in their hydrogen bonding availability is markedly visible in the CoMSIA hydrogen bond contour maps (for comparison see Figures 4C and 4D). There is a significant difference between Class I and Class II molecules in the large green area in the steric contour maps of both CoMSIA and the CoMFA (see also Supporting Figure 5). For Class I compounds the green area in this part of the structures is much smaller, and so it could signify a tighter contact with the receptor binding site.
(2.99 MB TIF)

Figure S5 Experimental versus CoMSIA predicted biological activities (−log ED_50_) for training set of Class II JH agonists. The significant difference between Class I and Class II molecules in the large green area in the steric contour maps of both CoMFA and the CoMSIA indicates that more bulky substituents in these regions will enhance the biological activity in Class II compounds. This might lead us to presume that there is a bigger or more flexible binding-site cavity surrounding this region in the agonists. The CoMSIA and also CoMFA generated steric and electrostatic contour maps have the potential to indicate the shape and surface requirements of the JH binding protein cavity, the putative JH-receptor. From this, we can infer that the receptor cavity must have charged residues lengthwise along its borders and negatively charged or neutral residues in its middle.
(2.99 MB TIF)

Experimental Procedure S1 (0.05 MB DOC)

Table S1 (0.24 MB DOC)

Table S2 (0.03 MB DOC)

Table S3 (0.02 MB DOC)

Table S4 (0.02 MB DOC)

Table S5 (0.02 MB DOC)

References S1 (0.04 MB DOC)
