# Supplementary figures and images for: Molecular Determinants of Juvenile Hormone Action as Revealed by 3D QSAR Analysis in Drosophila
Source: PLoS One. 2009 Jun 23;4(6):e6001. doi: 10.1371/journal.pone.0006001 (PMC2696086; doi:10.1371/journal.pone.0006001)

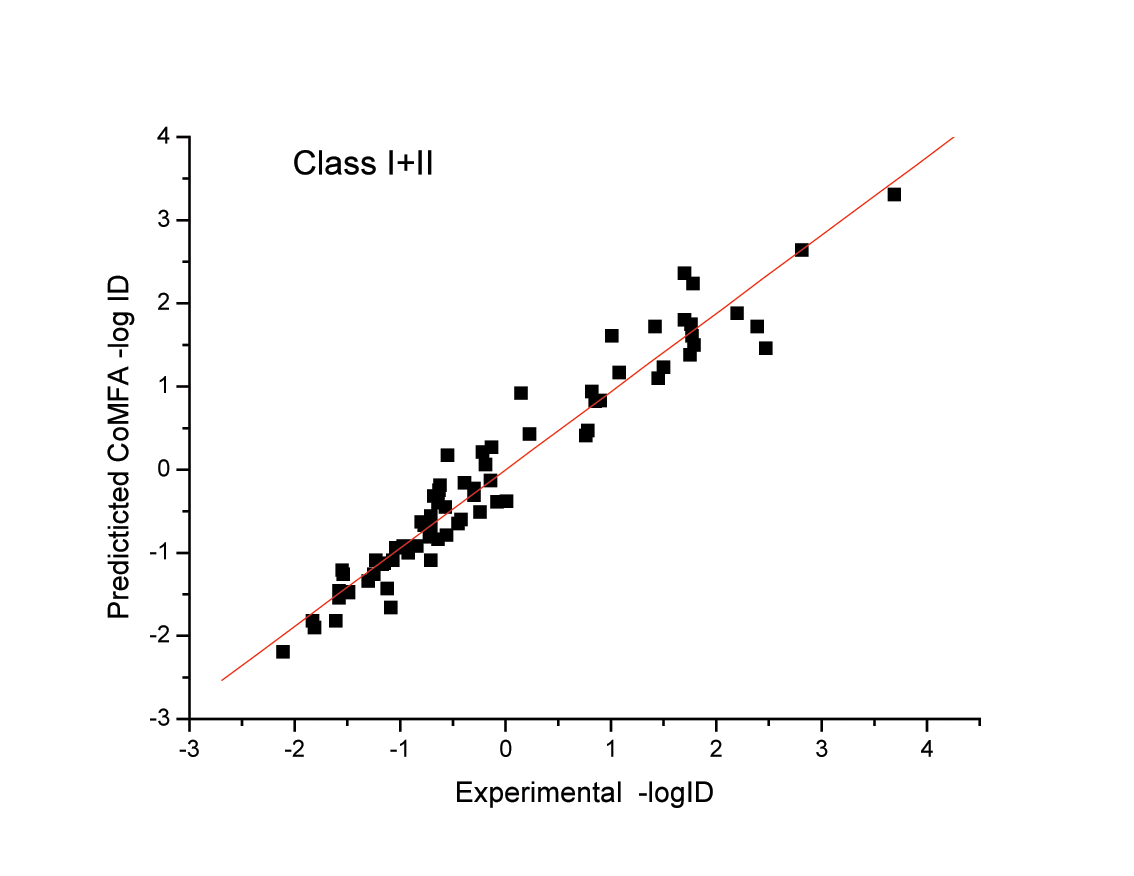

Supplement: Figure S1 — Comparative plot of experimental versus CoMFA predicted biological activities (−log ED50) of common training set (Class I+II) of 76 JH agonists. Despite being structurally diverse, most active compounds in Drosophila share some common features, i.e. an electronegative atom (oxygen or nitrogen) at one end of the molecule and electronegative atom (epoxy oxygen) or electron rich moiety (oxyphenyl group) on the molecule's opposite end (see compounds 1–3, 14–17, 19, 81–86). Nonetheless, the terpenoid and rigid phenoxy structures have very different chemical reactivity, atom charges and abilities in forming hydrogen bonds or electrostatic interactions. Indeed, this was one major reason to divide the complete training set into two classes. The oxygen in phenoxyphenol group of Class II compounds is sterically hindered by benzene rings that makes the phenoxyphenol oxygen poorly reactive for intermolecular hydrogen bonding, while the oxygen within an epoxy moiety of Class I compounds can easily provide electron pairs for H-bonding or for other electrostatic interactions. The difference between Class I and II analogs is reflected also in their negative charge distribution. In the Class I structures it is concentrated near electronegative, ether or epoxy oxygen whereas in Class II structures it is localized to the phenyl rings. Indeed, a similar protocol for subdividing compounds into two chemotypes for QSAR analyses was published recently for COX-2 inhibitors [41] and steroid hormones to reflect the unusual conformational adaptation of nuclear receptor ligand binding domains to agonist variety [42], [43]. Furthermore, the presence of an electron deficient moiety in the middle of the JH agonist molecule is essential for the very high biological activity seen in some synthetic JH agonists but not observed in natural JH (blue and cyan polyhedra regions in CoMFA and CoMSIA contour maps, respectively; see Figures 3 and 4A, B). On the other hand, the steric CoMFA and CoMSIA contour [file pone.0006001.s001.tif]

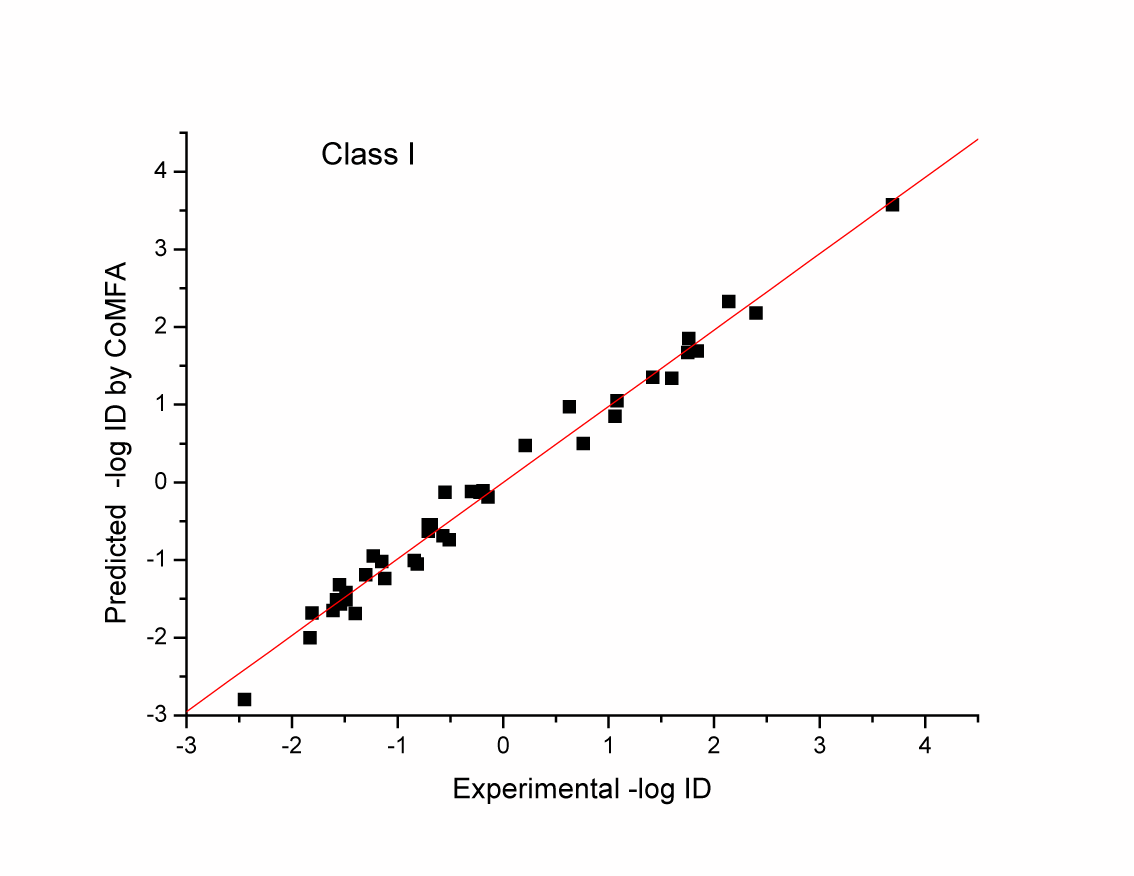

Supplement: Figure S2 — Graphical representation of observed versus CoMFA predicted biological activities (−log ED50) for training set of Class I JH agonists. (2.99 MB TIF) [file pone.0006001.s002.tif]

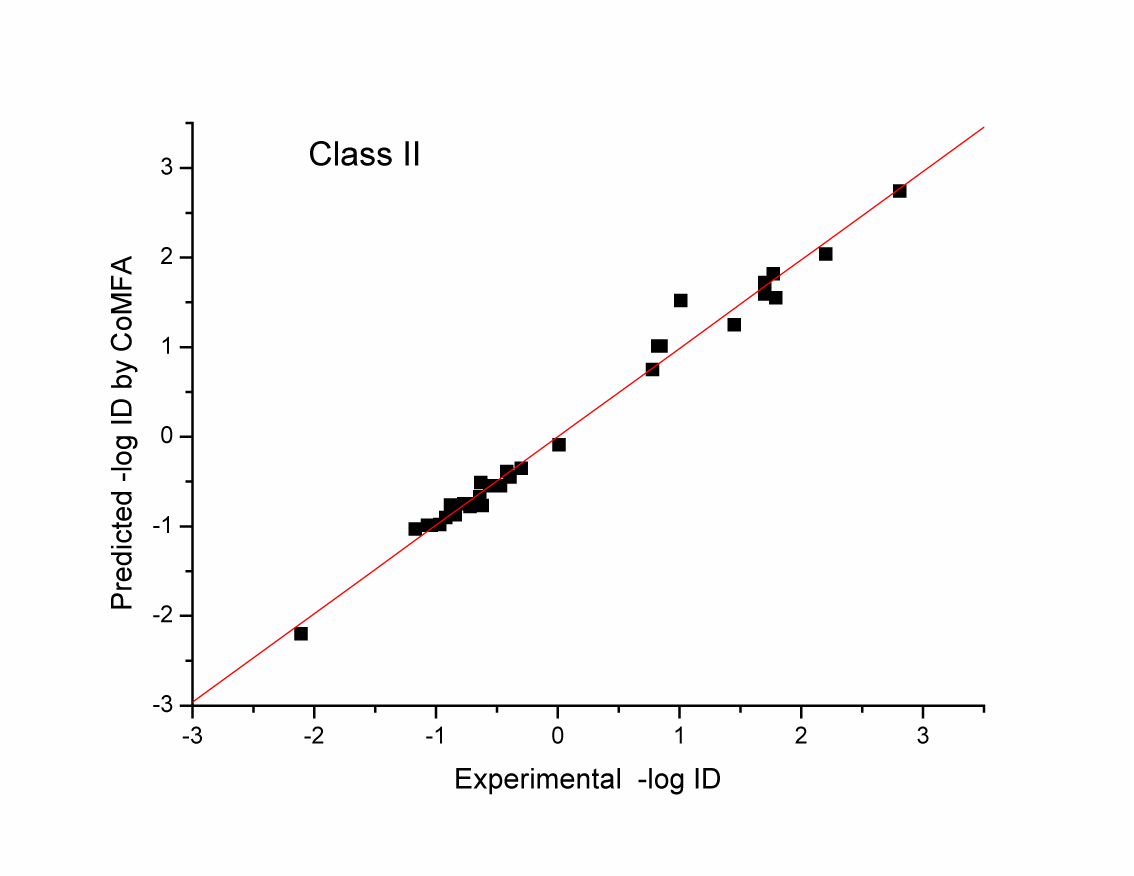

Supplement: Figure S3 — Correlation between experimental and CoMFA predicted biological activities (−log ED50) for training set of Class II JH agonists. (2.99 MB TIF) [file pone.0006001.s003.tif]

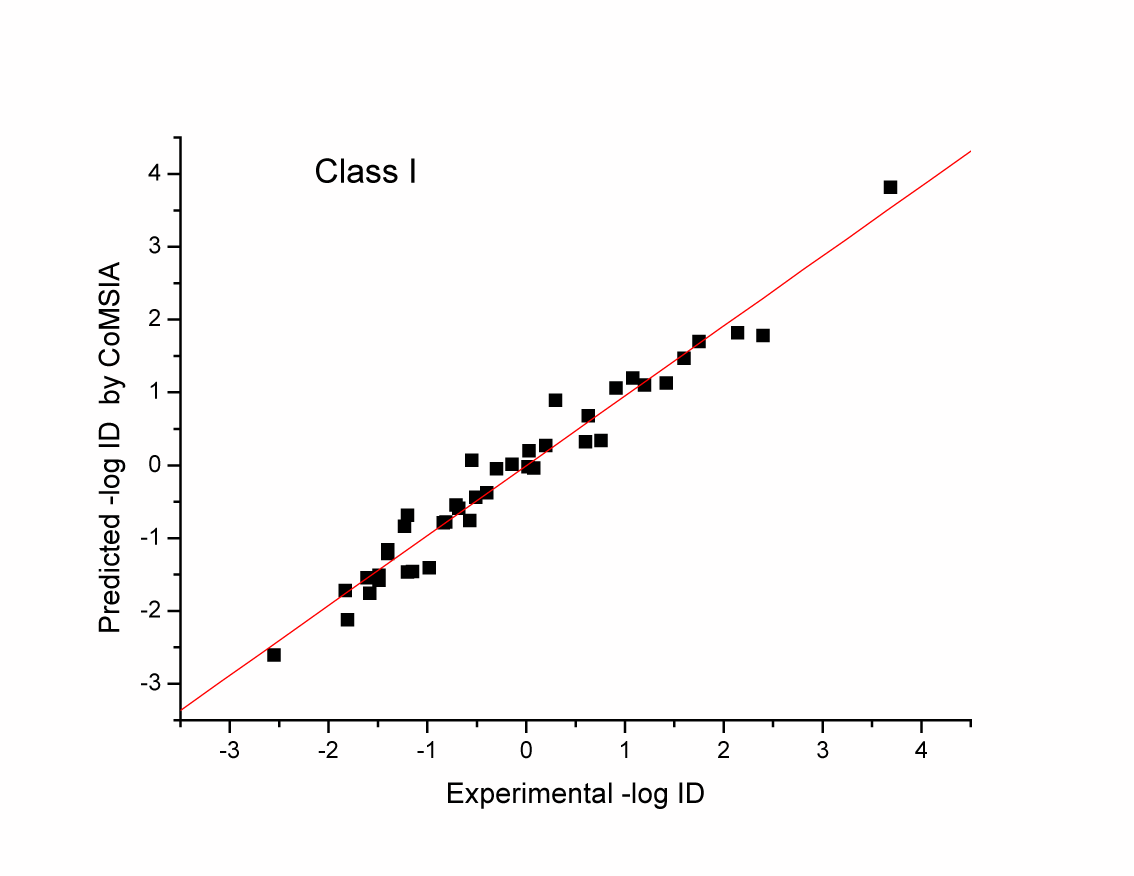

Supplement: Figure S4 — Graphical representation of observed versus CoMSIA predicted biological activities (−log ED50) for training set of Class I JH agonists. The difference between Class I and Class II agonists in their hydrogen bonding availability is markedly visible in the CoMSIA hydrogen bond contour maps (for comparison see Figures 4C and 4D). There is a significant difference between Class I and Class II molecules in the large green area in the steric contour maps of both CoMSIA and the CoMFA (see also Supporting Figure 5). For Class I compounds the green area in this part of the structures is much smaller, and so it could signify a tighter contact with the receptor binding site. (2.99 MB TIF) [file pone.0006001.s004.tif]

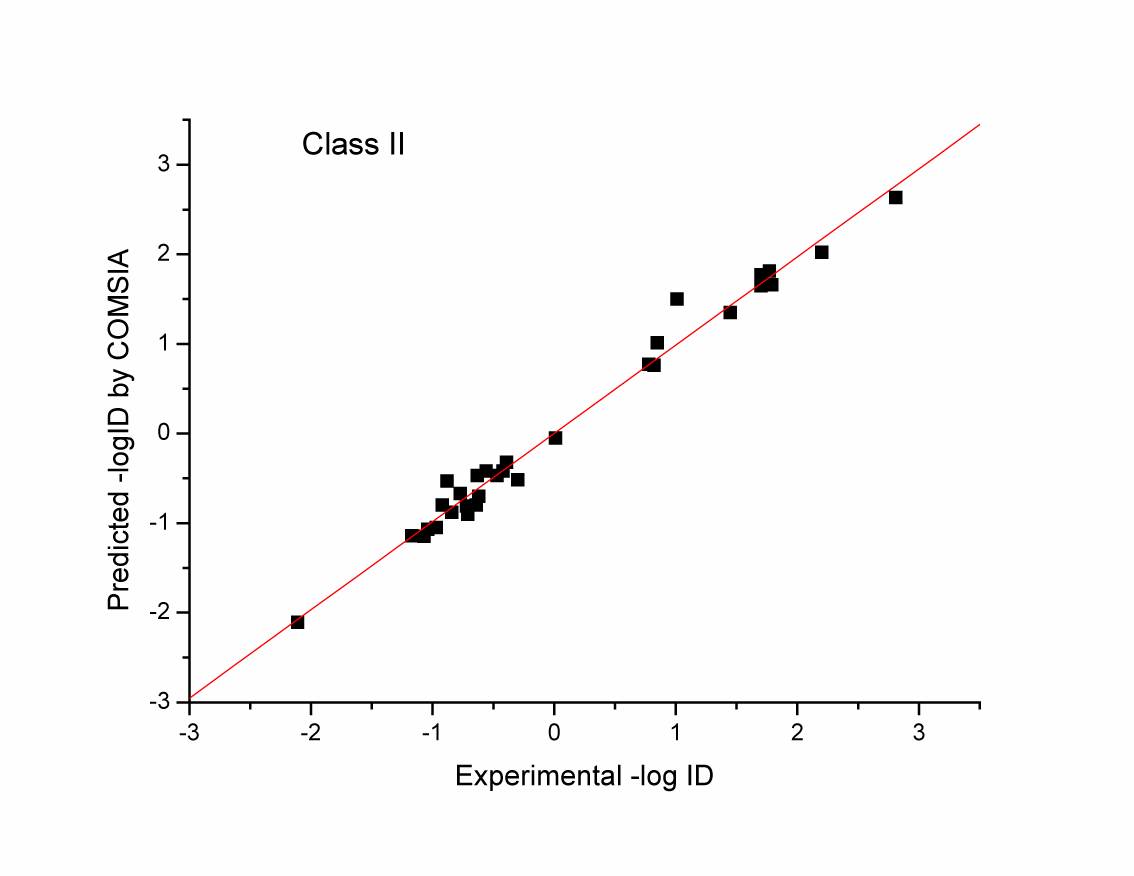

Supplement: Figure S5 — Experimental versus CoMSIA predicted biological activities (−log ED50) for training set of Class II JH agonists. The significant difference between Class I and Class II molecules in the large green area in the steric contour maps of both CoMFA and the CoMSIA indicates that more bulky substituents in these regions will enhance the biological activity in Class II compounds. This might lead us to presume that there is a bigger or more flexible binding-site cavity surrounding this region in the agonists. The CoMSIA and also CoMFA generated steric and electrostatic contour maps have the potential to indicate the shape and surface requirements of the JH binding protein cavity, the putative JH-receptor. From this, we can infer that the receptor cavity must have charged residues lengthwise along its borders and negatively charged or neutral residues in its middle. (2.99 MB TIF) [file pone.0006001.s005.tif]
