# Supplementary material for: Molecular Determinants of Juvenile Hormone Action as Revealed by 3D QSAR Analysis in Drosophila
Source: PLoS One. 2009 Jun 23;4(6):e6001. doi: 10.1371/journal.pone.0006001 (PMC2696086; doi:10.1371/journal.pone.0006001)
Supplement: Experimental Procedure S1 — (0.05 MB DOC) [file pone.0006001.s006.doc]

# Supporting Information

## Supporting Experimental Procedures

***Drosophila* culture and hormonal treatment of flies**.

All tests were performed on a wild-type strain *Oregon R* of *Drosophila* *melanogaster* (from Bloomington Stock Center, Indiana University, USA). Flies were cultured in 50 ml bottles at 23°C on ~10 ml of agar-yeast-cornmeal-molasses medium [1] with the addition of nipagin to inhibit mold growth.

JH and its analogs (listed in Supporting Table 1) were prepared freshly every day by dissolving in acetone (and never stored) and applied topically in 0.5 μl volume on the surface of late wandering 3rd instar larvae (cca 8-12 hr prior to pupariation), according to Ashburner [2]. All compounds were tested at starting dose of 1 μg per animal, and subsequently tested at more diluted concentrations. If compound displayed low or no activity at 1 μg/animal, doses were gradually increased to 10 μg/animal. Each concentration of every evaluated compound was tested on 200 larvae, and in triplicate. Glassware and plasticware used in these experiments was treated with 20% PEG 2000, extensively rinsed and autoclaved to prevent non-specific binding of JH. Undissolved compounds were stored in sealed glass tubes under nitrogen or argon atmosphere at -20°C. Due to various sources and origin of compounds, all JH and JH analogs in this study were first tested for their biological activity in well-defined bioassays on *Galleria* *mellonella* [3-5], *Tenebrio* *molitor* and *Pyrrhocoris* *apterus* [6]. Purity and identity of numerous terpenic (Class I) compounds [7-15] and selected compounds of Class II category [16-23] was verified by 2D NMR using Varian 500 MHz Unity Inova high-field NMR Spectrometer [24-25].

Although it was known that juvenile hormone in *Drosophila* and other related Cyclorrhaphous dipterans causes complex set of morphogenetic effects (perturbation of abdominal bristle differentiation including shortened or completely missing bristles, defects in abdominal cuticle pigmentation, misrotation of male genitalia, disruption of metamorphosis of the nervous and muscular systems, inhibition of eclosion) most tests of compounds for JH activity in different insect species have relied on using inhibition of eclosion as simplest parameter to evaluate their biological effectiveness. Since adult eclosion of many insects can be inhibited also by numerous non-hormonal chemicals due to non-specific and generally toxic effects, we chose to evaluate JH-activity of each compound by taking in account a specific, unambiguous morphogenetic effect. For purposes of this study, the ED_50_ was expressed as micrograms of the compound causing macrochaeteless and microchaeteless tergites in abdomens of 50% of the animals.

**Light and scanning electron microscopy**.

Pupal or adult cuticles were excised, fixed in 4% formaldehyde + 50% acetic acid, rinsed in ethanol, and delipidated in 1N KOH at 90°C for 5 min. After washing in saline, cuticles were dehydrated in ethanol, cleared in xylene and mounted in Euparal (Chroma GmbH.) or Entellan (E. Merck GmbH.). Alternatively, cuticles were fixed in glycerol-acetic acid (1:4), mounted and cleared overnight in Hoyer’s medium at 60°C [26]. These two techniques gave comparable results.

Preparation of adult flies for scanning electron microscopy was done as we described previously [27] with modifications for pupae and pharate adults as follows [28]. Hand-dissected pupae, pharate adults or eclosed adults if clean were fixed either in 3% glutaraldehyde and postfixed in 2% osmium tetroxide or directly fixed in 2% osmium tetroxide for a minimum of 24 hr. If the surface of dissected pupae or pharate adults was contaminated by dirt (originating mostly from pupal cuticle and/or exuvial fluid), animals were treated as follows: before fixation, animals were washed three times briefly in 15% Triton X-100 plus 5% Tween 20 and subsequently treated with 0.1 units of chitinase (Sigma GmbH.) and 0.2 units of β-*N*-acetylglucosaminidase (Oxford GlycoSciences Ltd.) for 1 to 3 hr at 37°C. This removed unwashable remnants of dried exuvial fluid. After dehydration through an ascending series of ethanol, animals were critical-point dried using hexamethyldisilazane (Sigma GmbH.) as described by Nation [29], and mounted on aluminum stubs. Samples were then sputter-coated with platinum or gold-palladium in a Balzers SCD-030, and viewed in a field emission electron source Hitachi S-800 scanning electron microscope operating at 10 kV.

**Computational analysis**.

Unless otherwise stated all the computational work was performed on Silicon Graphics Origin 2000 (R10000) and O_2_ (R10000) servers running under Irix 6.5.8 operating system.

**Structure building**

The 3D structures of the entire set of these compounds were built using the Sketch option in Sybyl 6.8 molecular modeling software (Tripos Co., St. Louis, MO, USA) on Silicon Graphics workstation and fully energetically minimized using the standard Tripos force field, with a 0.05 kcal/mol energy gradient convergence criterion and a distance-dependent dielectric constant. The systemic conformational search with a 30.0 angle increment was used to find local energy minimum conformations for all studied compounds. In addition, a random search routine was employed for finding the low energy conformations of structures with more than 3 rotable bonds. All generated low energy conformations were fully reoptimized with the AM1 semiempirical quantum chemical method which was also used for partial charges calculation. Resulting congener structures were refined against the X-ray or NMR data of structurally highly related compounds obtained from Cambridge Crystallographic Database. JH and some of its agonists are known to form chiral mixtures [30]. Biological activity of many enantiomer forming compounds in general appear to play an important role in enzymatic reactions, stereoselective degradation and other mechanisms [31-33]. Importantly, X-ray structural studies have shown that biologically less active enantiomers of nuclear hormone receptor ligands are also able to bind receptor pocket and adopt bioactive conformation as more active enantiomers, although this conformation is energetically less favored [34-36]. Thus, enantioselective isomers and chiral centers of the molecules were considered where appropriate according to the descriptors for 3D QSAR analysis as described by Golbraikh *et* *al*. [37], Paier *et al*. [38], and Kovatcheva *et* *al*. [39]. In CoMSIA we used the Gaussian function for the distance dependence between the probe atom and the molecule atoms to avoid some of the inherent deficiencies arising from the functional form of the Lennard-Jones and Coulomb potentials [40].

**Supporting References**

1. Ransom R (1982) A Handbook of Drosophila Development. Amsterdam and New York: Elsevier

Biomedical Press.

2. Ashburner M (1970) Effects of juvenile hormone on adult differentiation of *Drosophila*

*melanogaster*. Nature 227: 187-189.

3. Jarolím V, Hejno K, Sehnal F, Šorm F (1969) Natural and synthetic materials with insect

hormones activity. 8. Juvenile activity of the farnesane-type compounds on *Galleria* *mellonella*.

Life Sci 8: 831-841.

4. Benz G (1971) Failure to demonstrate sterilans effect of juvenile hormone mimetics in *Pieris*

*brassicae* and *Galleria mellonella*. Experientia 27: 581-582.

5. DeLoof A, Van de Veire M (1972) Time saving improvements in the *Galleria* bioassay for

juvenile hormone. Experientia 28: 366-367.

6. Sláma K, Romaňuk M, Šorm F (1974) Insect Hormones and Bioanalogues. Wien, Heidelberg

and New York: Springer Verlag.

7. Bowers ES (1969) Juvenile hormone: activity of aromatic terpenoid ethers. Science 164: 323-325.

8. Zaoral M, Sláma K (1970) Peptides with juvenile hormone activity. Science 170: 92-93.

9. Schwarz M, Redfern RE, Waters RM, Wakabayashi N, Sonnet PE (1971) Compounds related to

juvenile hormone. X. Activity of selected arylterpenoid compounds on *Tenebrio molitor* L. and

*Oncopeltus fasciatus* (Dallas). Life Sci II 10: 1125-1132.

10. Henrick CA, Staal GB, Siddall JB (1973) Alkyl 3,7,11-trimethyl-2,4-dodecadienoates, a new

class of potent insect growth regulators with juvenile hormone activity. J Agric Food Chem 21:

354-359.

11. Brieger G, Ellis RF (1975) Terpenoid ethers as juvenile hormone analogs. J Agric Food Chem

23: 335-337.

12. Henrick CA, Staal GB, Siddall JB (1973) Alkyl 3,7,11-trimethyl-2,4-dodecadienoates, a new

class of potent insect growth regulators with juvenile hormone activity. J Agric Food Chem

21: 354-359.

13. Henrick CA, Willy WE, Garcia BA, Staal GB (1975) Insect juvenile hormone activity of the

stereoisomers of ethyl 3,7,11-trimethyl-2,4-dodecadienoate. J Agric Food Chem 23: 396-400.

14. Henrick CA, Willy WE, Staal GB (1976) Insect juvenile hormone activity of alkyl (2E,4E)-

3,7,11-trimethyl-2,4-dodecadienoates. Variations in the ester function and in the carbon chain.

J Agric Food Chem 24: 207-218.

15. Sehnal F, Ždárek J (1976) Action of juvenoids on the metamorphosis of cyclorrhaphous diptera.

J Insect Physiol 22: 673-682.

16. Kramer KJ, McGregor HE, Mori K (1979) Susceptibility of stored product insects to pyridyl

ether analogues of juvenile hormone. J Agric Food Chem 27: 1215-1217.

17. Nakayama A, Iwamura H, Niwa A, Nakagawa Y, Fujita T (1985) Development of insect juvenile

hormone active oxime *o*-ethers and carbamates. J Agric Food Chem 33: 1034-1041.

18. Hatakoshi M, Agui N, Nakayama I (1986) 2-[1-Methyl-2-(4-phenoxy-phenoxy)ethoxy]-pyridine

as a new insect juvenile hormone analog induction of supernumerary larvae in *Spodoptera* *litura*

(*Lepidoptera*: *Noctuidae*). Appl Entomol Zool 21: 351-353.

19. Masner P, Angst M, Dorn S (1987) Fenoxycarb, an insect growth regulator with juvenile

hormone activity: A candidate for *Heliothis* *virescens* (F.) control on cotton. Pestic Sci 18: 89-94.

20. Niwa A, Iwamura H, Nakagawa Y, Fujita T (1989) Development of (phenoxyphenoxy)- and

(benzylphenoxy)-propyl ethers as potent insect juvenile hormone mimetics. J Agric Food Chem

37: 462-467.

21. Niwa A, Iwamura H, Nakagawa Y, Fujita T (1989) Development of (phenoxyphenoxy)- and

(benzylphenoxy)propyl ethers as potent insect juvenile hormone mimetics J Agric Food Chem

37: 467-472.

22. Henrick CA (1995) Juvenoids. In: Godfrey CRA, editor. Agrochemicals from Natural Products.

New York and Basel: Marcel Dekker Press. pp. 147-213.

23. Wimmer Z, Rejzek M, Zarevúcka M, Kuldová J, Hrdý I et al. (1997) A series of bicyclic insect

juvenile hormone analogs of Czech origin: Twenty years of development. J Chem Ecol 23: 605-628.

24. Maxwell RA, Anderson RJ, Schooley DA (2002) Simultaneous preparation of both enantiomers

of juvenile hormones labeled at C-10 with tritium at high specific activity. Anal Biochem 305:

40-48.

25. Ichikawa A, Ono H, Furuta K, Shiotsuki T, Shinoda T (2007) Enantioselective separation of

racemic juvenile hormone III by normal-phase high-performance liquid chromatography and

preparation of [^2^H_3_]juvenile hormone III as an internal standard for liquid chromatography-mass

spectrometry quantification. J Chromatogr A 1161: 252-260.

26. van der Meer JM (1977) Optical clean and permanent whole mount preparation for phase-contrast

microscopy of cuticular structures of insect. larvae. Dros Inf Serv 52: 160-161.

27. Yun B, Farkaš R, Lee K, Rabinow L (1994) The *Doa* locus encodes a member of a new protein

kinase family and is essential for eye and embryonic development in *Drosophila melanogaster*.

Genes Dev 8: 1160-1173.

28. Beňo M, Liszeková D, Farkaš R (2007) Processing of soft pupae and uneclosed pharate adults

of *Drosophila* for scanning electron microscopy. Micros Res Tech 70: 1022-1027.

29. Nation JL (1983) A new method using hexamethyldisilazane for preparation of soft insect tissues

for scanning electron microscopy. Stain Technol 58: 347-351.

30. Prestwich GD, Wawrzenczyk C (1985) High specific activity enantiomerically enriched juvenile

hormones: synthesis and binding assay. Proc Natl Acad Sci USA 82: 5290-5294.

31. Cody JT, Valtier S, Nelson SL (2004) Amphetamine excretion profile following multidose

administration of mixed salt amphetamine preparation. J Anal Toxicol 28: 563-574.

32. Gadler P, Faber K (2007) New enzymes for biotransformations: microbial alkyl sulfatases

displaying stereo- and enantioselectivity. Trends Biotechnol 25: 83-88.

33. Nillos MG, Rodriguez-Fuentes G, Gan J, Schlenk D (2007) Enantioselective

acetylcholinesterase inhibition of the organophosphorous insecticides profenofos, fonofos, and

crotoxyphos. Environ Toxicol Chem 26: 1949-1954.

34. Klaholz BP, Renaud JP, Mitschler A, Zusi C, Chambon P et al. (1998) Conformational

adaptation of agonists to the human nuclear receptor RAR gamma. Nature Struct Biol 5:

199-202.

35. Klaholz BP, Mitschler A, Belema M, Zusi C, Moras D (2000) Enantiomer discrimination

illustrated by high-resolution crystal structures of the human nuclear receptor hRARgamma.

Proc Natl Acad Sci USA 97: 6322-6327.

36. Klaholz BP, Mitschler A, Moras D (2000) Structural basis for isotype selectivity of the human

retinoic acid nuclear receptor. J Mol Biol 302: 155-170.

37. Golbraikh A, Bonchev D, Tropsha A (2001) Novel chirality descriptors derived from molecular

topology. J Chem Inf Comput Sci 41: 147-158.

38. Paier J, Stockner T, Steinreiber A, Faber K, Fabian WM (2003) Enantioselectivity of epoxide

hydrolase catalysed oxirane ring opening: a 3D QSAR study. J Comput Aided Mol Des 17: 1-11.

39. Kovatcheva A, Golbraikh A, Oloff S, Feng J, Zheng W et al. (2005) QSAR modeling of

datasets with enantioselective compounds using chirality sensitive molecular descriptors.

SAR QSAR Environ Res 16: 93-102.

40. Cramer RD, Patterson DE, Bunce JD (1989) Recent advances in comparative molecular field

analysis (CoMFA). Prog Clin Biol Res 291: 161-165.

41. Kim HJ, Chae CH, Yi KY, Park KL, Yoo SE (2004) Computational studies of COX-2

inhibitors: 3D-QSAR and docking. Biooorg Med Chem 12: 1629-1641.

42. Togashi M, Borngraeber S, Sandler B, Fletterick RJ, Webb P et al. (2005) Conformational

adaptation of nuclear receptor ligand binding domains to agonists: potential for novel approaches

to ligand design. J Steroid Biochem Mol Biol 93: 127-137.

43. Wheelock CE, Nakagawa Y, Harada T, Oikawa N, Akamatsu Met al. (2006) High-throughput

screening of ecdysone agonists using a reporter gene assay followed by 3-D QSAR analysis of the

molting hormonal activity. Biooorg Med Chem 14: 1143-1159.
