# Supplementary material for: Molecular Determinants of Juvenile Hormone Action as Revealed by 3D QSAR Analysis in Drosophila
Source: PLoS One. 2009 Jun 23;4(6):e6001. doi: 10.1371/journal.pone.0006001 (PMC2696086; doi:10.1371/journal.pone.0006001)
Supplement: Table S1 — (0.24 MB DOC) [file pone.0006001.s007.doc]

**Supporting Table 1.**  **Complete list of JH agonists used in this study and their biological activities**. The biological activity (ED_50_) is expressed in μg of the compound per animal, then it is converted to nmol per animal, and finally to -log of nmol values that are used for CoMFA and CoMSIA computations. Compounds 1 - 50 represent Class I agonists, whereas compounds 51 - 86 represent Class II agonists. Chemical names are according to IUPAC nomenclature, with trivial or commercial names in the parentheses where appropriate.

| **N^o^** | Compound | **ED_50_ [μg]** | **ED_50_ [nM]** | **-log** **ED_50_ [nM]** |
| --- | --- | --- | --- | --- |
| **1** |  | 0.0100 | 0.0375 | 1.4259 |
| **2** |  | 1.0000 | 3.5695 | -0.5526 |
| **3** |  | 0.0500 | 0.1699 | 0.7698 |
| **4** |  | 0.5000 | 1.8922 | -0.2769 |
| **5** |  | 1.5000 | 5.2418 | -0.7194 |
| **6** |  | 2.5000 | 8.9482 | -0.9517 |
| **7** |  | 0.1150 | 0.4070 | 0.3904 |
| **8** |  | 0.0080 | 0.0317 | 1.4989 |
| **9** |  | 0.00005 | 0.0002 | 3.6989 |
| **10** |  | 5.0000 | 20.1499 | -1.3042 |
| **11** |  | 0.0250 | 0.0873 | 1.0589 |
| **12** |  | 2.0000 | 6.9302 | -0.8407 |
| **13** |  | 2.5000 | 8.3166 | -0.9199 |
| **14** |  | 0.0050 | 0.0177 | 1.7520 |
| **15** |  | 0.0010 | 0.0034 | 2.4685 |
| **16** |  | 0.1250 | 0.5266 | 0.2785 |
| **17** |  | 0.00125 | 0.0040 | 2.3979 |
| **18** |  | 4.0000 | 13.5057 | -1.1305 |
| **19** |  | 5.0000 | 16.8827 | -1.2274 |
| **20** |  | 0.0050 | 0.0167 | 1.7773 |
| **21** |  | 0.2000 | 0.7515 | 0.1207 |
| **22** |  | 0.4000 | 1.3506 | -0.1322 |
| **23** |  | 0.5000 | 1.5471 | -0.1895 |
| **24** |  | 0.5000 | 1.7783 | -0.2500 |
| **25** |  | 1.0000 | 16.8827 | -1.2274 |
| **26** |  | 1.5000 | 5.1339 | -0.7104 |
| **27** |  | 5.0000 | 17.8475 | -1.2515 |
| **28** |  | 1.0000 | 2.8411 | -0.4535 |
| **29** |  | 10.0000 | 39.9712 | -1.6017 |
| **30** |  | 10.0000 | 40.8163 | -1.6108 |
| **31** |  | 10.0000 | 38.1402 | -1.5814 |
| **32** |  | 10.0000 | 38.1402 | -1.5814 |
| **33** |  | 10.0000 | 35.4358 | -1.5494 |
| **34** |  | 10.0000 | 31.2275 | -1.4945 |
| **35** |  | 0.0040 | 0.0144 | 1.8416 |
| **36** |  | 0.0100 | 0.0364 | 1.4380 |
| **37** |  | 5.0000 | 16.3323 | -1.2130 |
| **38** |  | 10.0000 | 34.1157 | -1.5329 |
| **39** |  | 10.0000 | 31.0414 | -1.4919 |
| **40** |  | 4.0000 | 14.1548 | -1.1509 |
| **41** |  | 1.0000 | 3.7512 | -0.5742 |
| **42** |  | 4.0000 | 13.1501 | -1.1189 |
| **43** |  | 1.2500 | 5.1200 | -0.7092 |
| **44** |  | 1.2500 | 4.8053 | -0.6817 |
| **45** |  | 5.0000 | 18.9315 | -1.2771 |
| **46** |  | 0.0050 | 0.0172 | 1.7645 |
| **47** |  | 0.5000 | 1.6439 | -0.2158 |
| **48** |  | 0.3500 | 1.2063 | -0.0814 |
| **49** |  | 7.5000 | 22.3751 | -1.3497 |
| **50** |  | 0.4000 | 1.3880 | -0.1423 |
| **51** |  | 5.0000 | 14.9701 | -1.1752 |
| **52** |  | 5.0000 | 14.0619 | -1.1480 |
| **53** |  | 5.0000 | 11.7799 | -1.0711 |
| **54** |  | 0.0100 | 0.0356 | 1.4485 |
| **55** |  | 0.0400 | 0.1423 | 0.8467 |
| **56** |  | 0.0013 | 0.0041 | 2.3872 |
| **57** |  | 0.0500 | 0.1663 | 0.7791 |
| **58** |  | 0.0500 | 0.1523 | 0.8171 |
| **59** |  | 2.0000 | 5.9667 | -0.7757 |
| **60** |  | 0.8500 | 2.6061 | -0.4159 |
| **61** |  | 5.0000 | 17.8475 | -1.2515 |
| **62** |  | 0.8000 | 2.4487 | -0.3889 |
| **63** |  | 1.2000 | 3.9328 | -0.5947 |
| **64** |  | 1.0000 | 2.9837 | -0.4747 |
| **65** |  | 3.5000 | 10.9669 | -1.0401 |
| **66** |  | 1.4000 | 4.3455 | -0.6380 |
| **67** |  | 1.0000 | 3.1043 | -0.4919 |
| **68** |  | 1.4000 | 4.3867 | -0.6421 |
| **69** |  | 0.3000 | 0.9895 | 0.0045 |
| **70** |  | 1.1500 | 3.6146 | -0.5580 |
| **71** |  | 2.3000 | 6.9038 | -0.8391 |
| **72** |  | 1.8000 | 5.1409 | -0.7110 |
| **73** |  | 0.8500 | 2.4345 | -0.3864 |
| **74** |  | 1.5000 | 4.1192 | -0.6148 |
| **75** |  | 1.9000 | 5.2319 | -0.7186 |
| **76** |  | 1.0000 | 2.6583 | -0.4246 |
| **77** |  | 2.9000 | 7.6890 | -0.8858 |
| **78** |  | 3.2700 | 0.2426 | 0.6151 |
| **79** |  | 0.7500 | 1.9885 | -0.2985 |
| **80** |  | 1.6000 | 4.2422 | -0.6275 |
| **81** |  | 0.0020 | 0.0063 | 2.2006 |
| **82** |  | 0.0060 | 0.0198 | 1.7033 |
| **83** |  | 0.0060 | 0.0199 | 1.7011 |
| **84** |  | 0.0050 | 0.0162 | 1.7904 |
| **85** |  | 0.0055 | 0.0169 | 1.7721 |
| **86** |  | 0.0005 | 0.00156 | 2.8068 |

**1** = (*2E,6E*)-9-((*R*)3,3-Dimethyl-oxiranyl)-3,7-dimethyl-nona-2,6-dienoic acid methyl ester (JH-III, also known as methylepoxyfarnesoate)

**2** = (*2E,6E*)-9-((*2R*,*3S*)3-Ethyl-3-methyl-oxiranyl)-3,7-dimethyl-nona-2,6-dienoic acid methyl ester (JH-II)

**3** = (*2E,6E*)-7-Ethyl-9-((*2R*,*3S*)3-ethyl-3-methyl-oxiranyl)-3-methyl-nona-2,6-dienoic acid methyl ester (JH-I)

**4** = (*2E,6E*)-9-((*R*)3,3-Dimethyl-oxiranyl)-3,7-dimethyl-nona-2,6-dienoic acid (JH-III acid)

**5** = (*2E,6E*)-(*S*)-10,11-Dihydroxy-3,7,11-trimethyl-dodeca-2,6-dienoic acid methyl ester (JH-III diol)

**6** = (*2E,6E*)-(*S*)-10,11-Dihydroxy-3,7,11-trimethyl-dodeca-2,6-dienoic acid (JH-III acid diol)

**7** = (E)-5-{3-2-[((6*R*,*7S*,*10R*)-3,3-Dimethyl-oxiranyl)-ethyl]-3-methyl-oxiranyl}-3-methyl-pent-2-enoic acid methyl ester; Methyl-6,7;10,11-bisepoxy-3,7,11-trimethyl-(*E*)-dodecenoate (JH-III bisepoxide, JHB_3_)

**8** = (*E*)-(*S*)-3,7,11-Trimethyl-dodeca-2,10-dienoic acid methyl ester

**9** = (*E*)-4-[4-((*2R*)-3,3-Dimethyl-oxiranyl)-(*2S*)-2-methyl-butoxy]-3-methyl-but-2-enoic acid methyl ester (SJ-68 oxid)

**10** = (*2E,6E*)-3,7,11-Trimethyl-dodeca-2,6,10-trienoic acid methyl ester (methylfarnesoate)

**11** = (*3E,5E,7E*)-5,8,12,12-Tetramethyl-trideca-3,5,7-trienoic acid methyl ester

**12** = (*E*)-(*R*)-11-Chloro-3,7,11-trimethyl-dodec-2-enoic acid methyl ester

**13** = (*E*)-(*R*)-(11)-Chloro-3,7,11-trimethyl-dodec-2-enoic acid ethyl ester

**14** = (*E*)-9-((*R*)-3,3-Dimethyl-oxiranyl)-3,7-dimethyl-non-2-enoic acid ethyl ester

**15** = (*2E,4E*)-(*R*)-11-Methoxy-3,7,11-trimethyl-dodeca-2,4-dienoic acid ethyl ester (methoprene; altosid; ZR-515)

**16** = Tioethyl-(*2E,4E*)-(*R*)-11-methoxy-3,7,11-trimethyl-2,4-dodecadienoate (triprene; ZR-619)

**17** = (*2E,4E*)-(*R*)-11-Methoxy-3,7,11-trimethyl-dodeca-2,4-dienoic acid isopropyl ester

**18** = (*E*)-(*R*)-3,7,11,11-Tetramethyl-tridec-2-enoic acid ethyl ester

**19** = Ethyl-((*1E,5E*)-10-methoxy-2,6,10-trimethylundeca-1,5-dienyloxymethyl)-amine

**20** = (*E*)-(*R*)-11-Methoxy-3,7,11-trimethyl-dodec-2-enoic acid ethyl ester

**21** = (*2E,4E*)-(*R*)-3,7,11-Trimethyl-dodeca-2,4-dienoic acid ethyl ester (hydroprene; altozar; ZR-512)

**22** = (*2E,4E*)-(*R*)-11-Methoxy-3,7,11-trimethyl-dodeca-2,4-dienoic acid ethylamide

**23** = (*2E,4E*)-(*R*)-11-Methoxy-3,7,11-trimethyl-dodeca-2,4-dienoic acid diethylamide (ZR-618)

**24** = (*4E,6E*)-(*R*)-13-Methoxy-5,9,13-trimethyl-tetradeca-4,6-dien-3-ol

**25** = (*E*)-(*R*)-3,7,11-Trimethyl-dodec-2-enoic acid ethyl ester

**26** = (*2E,6E,10Z*)-7-Ethyl-3,11-dimethyl-trideca-2,6,10-trienoic acid ethyl ester

**27** = (*2E,4E*)-(*R*)-3,7,11-Trimethyl-dodeca-2,4-dienoic acid prop-2-ynyl ester (kinoprene; ZR-777)

**28** = 2-((*E*)-(*8R*,*9S*)-9-Ethoxy-4,8-dimethyl-dec-3-enyl)-2-methyl-(*2R*,*3S*)-cyclopropanecarboxylic acid isopropyl ester (ZR-4429)

**29** = (*2S*,*7R*)-2-Ethyl-3,8,12-trimethyl-tridec-1-ene

**30** = 2-Ethyl-4,8,12-trimethyl-trideca-1,3,7,11-tetraene

**31** = (*6E,10E*)-2,6,10,14-Tetramethyl-pentadeca-2,6,10-triene

**32** = (*6E,10E*)-2,6,10-Trimethyl-hexadeca-2,6,10-triene

**33** = (*6R*,*10S*)-2,6,10,15-Tetramethyl-hexadecane

**34** = (*3R*,*8S*,*12S*)-2-Ethyl-3,8,12,16-tetramethyl-heptadec-1-ene

**35** = (*R*)-3-[5-(3-Ethyl-phenoxy)-3-methyl-pentyl]-2,2-dimethyl-oxirane

**36** = (*R*)-3-[(*E*)-5-(4-Ethyl-phenoxy)-3-methyl-pent-3-enyl]-2,2-dimethyl-oxiran

**37** = 3-[5-((*R*)-3,3-Dimethyl-oxiranyl)-3-methyl-pentyloxy]-benzoic acid methyl ester

**38** = (*R*)-2,2-Dimethyl-3-[3-methyl-5-(3-nitro-phenoxy)-pentyl]-oxirane

**39** = (*R*)-3-[5-(3-Ethoxymethoxy-phenoxy)-3-methyl-pentyl]-2,2-dimethyl-oxirane

**40** = (*R*)-3-[5-(4-Chloro-phenoxy)-3-ethyl-pentyl]-2,2-dimethyl-oxirane

**41** = (*R*)-3-{[(*E*)-4]-4-Chloro-phenoxy)}-3-methyl-but-3-enyl]-2,2-dimethyl oxirane

**42** = (*2R*,*3S*)-2-Ethyl-3-[-(*3S*)-3-ethyl-5-(4-ethyl-phenoxy)-pentyl]-2-methyl-oxirane (epofenonane)

**43** = 3-((*E*)-2,6-Dimethyl-hepta-1,5-dienyloxy)-benzaldehyde

**44** = 5-((*E*)-2,6-Dimethyl-hepta-1,5-dienyloxy)-benzo-[1,3]-dioxole

**45** = 5-[3-((*2R*)-3,3-Dimethyl-oxiranyl)-(*1S*)-1-methyl-propoxy]-benzo-[1,3]-dioxole

**46** = 5-[(*E*)-5-((*R*)-3,3-Dimethyl-oxiranyl)-3-methyl-pent-2-enyloxy]-benzo-[1,3]-dioxole

**47** = 5-[(*E*)-5-((*2R*,*3S*)-3-Ethyl-3-methyl-oxiranyl)-3-methyl-pent-2-enyloxy]-benzo-[1,3]-dioxole

**48** = 5-[(*E*)-4-((*2R*,*3S*)-3-Ethyl-3-methyl-oxiranyl)-2-methyl-but-1-enyloxy]-benzo-[1,3]-dioxole

**49** = 2-(2,2-Dimethyl-propionylamino)-3-methyl-pentanoic acid benzo-[1,3]-dioxol-5-yl amide

**50** = 3-[5-(Indan-4-yloxy)-3-(*S*)-methyl-pentyl]-(*3R*)-2,2-dimethyl-oxirane

**51** = (*S*)-4-[2-(2,2-Dimethyl-propyonylamino)-propionylamino]-benzoic acid ethyl ester

**52** = (*S*)-4-(2-{[(2-Chloro-propionylamino)-methyl]-amino}-propionylamino)-benzoic acid ethyl ester

**53** = (*S*)-4-{2-[3-(2,2,2-Trichloro-acetyl)-ureido]-propionylamino}-benzoic acid ethyl ester

**54** = [2-(4-(*R*)-sec-Butoxy-phenoxy)-ethyl]-carbamic acid ethyl ester

**55** = Ethyl-carbamic acid 2-(4-(*R*)-sec-butoxy-phenoxy)-ethyl ester

**56** = [2-(4-(*R*)-sec-Butylsulfanyl-phenoxy)-ethyl]-thiocarbamic acid S-ethyl ester

**57** = [2-(4-Phenoxy-phenoxy)-ethyl]-carbamic acid ethyl ester (fenoxycarb)

**58** = 1-[2-(*S*)-(3-(*R*)-Ethoxy-butoxy)-propyl]-4-phenoxy-benzene

**59** = (*E*)-3,7-Dimethyl-octa-2,6-dienoic acid 4-phenoxy-phenyl ester

**60** = [(4-Phenoxy-phenylcarbamoyl)-methyl]-carbamic acid tert-butyl ester

**61** = 1-[2-(4-Phenoxy-phenoxy)-ethyl]-1H-pyrazol

**62** = (*R*)-2-[1-Methyl-2-(4-phenoxy-phenoxy)-ethoxy]-thiazole

**63** = (*S*)-Carbonic acid ethyl ester 2-[4-(2-oxo-cyclopentylmethyl)-phenoxy]-ethyl ester

**64** = (*S*)-{2-[4-(2-Methoxy-cyclohexylmethyl)-phenyl]-ethyl}-carbamic acid ethyl ester

**65** = (*S*)-Ethyl-carbamic acid 2-[4-(2-hydroxy-cyclohexylmethyl)-phenoxy]-ethyl ester

**66** = (*S*)-Carbonic acid ethyl ester 2-[4-((*R*)-2-hydroxy-cyclohexylmethyl)-phenoxy]-ethyl ester

**67** = (*S*)-Carbonic acid ethyl ester 2-[4-((*S*)-2-hydroxy-cyclohexylmethyl)-phenoxy]-ethyl ester

**68** = (*S*)-Ethyl-carbamic acid 2-[4-(2-oxo-cyclohexylmethyl)-phenoxy]-ethyl ester

**69** = (*S*)-{2-[4-(2-Oxo-cyclohexylmethyl)-phenoxy]-ethyl}-carbamic acid ethyl ester

**70** = (*S*)-1-Ethyl-3-{2-[4-(2-oxo-cyclohexylmethyl)-phenoxy]-ethyl}-urea

**71** = (*S*)-{2-[4-(2-Oxo-cycloheptylmethyl)-phenoxy]-ethyl}-carbamic acid ethyl ester

**72** = (*R*)-Carbonic acid 2-[4-(1,4-dioxa-spiro-[4.4]-non-6-ylmethyl)-phenoxy]-ethyl ester ethyl ester

**73** = (*R*)-{2-[4-(1,4-Dioxa-spiro-[4,4]-non-6-ylmethyl)-phenoxy]-ethyl}-carbamic acid ethyl ester

**74** = (*S*)-Carbonic acid 2-[4-(1,4-dioxa-spiro-[4,5]-dec-6-ylmethyl)-phenoxy]-diethyl ester

**75** = (*S*)-Ethyl-carbamic acid 2-[4-(1,4-dioxa-spiro-[4,5]-dec-6-yl-methyl)-phenoxy]-ethyl ester

**76** = (*S*)-1-{2-[4-(1,4-Dioxa-spiro-[4,5]-dec-6-ylmethyl)-phenoxy]-ethyl}-3-ethyl-urea

**77** = {2-[4-(10-(*R*)-Methyl-1,4-dioxa-spiro-[4,5]-dec-6-(*S*)-yl-methyl)-phenoxy]-ethyl}-carbamic acid ethyl ester

**78** = (*S*)-{2-[4-(1,4-Dithia-spiro-[4,5]-dec-6-yl-methyl)-phenoxy]-ethyl}-carbamic acid ethyl ester

**79** = (*S*)-{2-[4-(1,5-Dioxa-spiro-[5,5]-undec-7-ylmethyl)-phenoxy]-ethyl}-carbamic acid ethyl ester

**80** = (*S*)-{2-[4-(1,4-Dioxa-spiro-[4,6]-undec-6-ylmethyl)-phenoxy]-ethyl}-carbamic acid ethyl ester

**81** = 2-[2-(4-Phenoxy-phenoxy)-ethoxy]-thiazole (ZR-10183)

**82** = 3-[(*E*)-3-(4-Phenoxy-phenoxy)-prop-1-enyl]-pyridine (ZR-10852)

**83** = 2-[3-(4-Phenoxy-phenoxy)-prop-1-ynyl]-pyridine (ZR-10797)

**84** = 2-[2-(4-Phenoxy-phenoxy)-ethoxy]-pyrimidine

**85** = 2-[2-(4-Phenoxy-phenoxy)-ethylsulfanyl]-pyrimidine (ZR-10131)

**86** = 2-[1-Methyl-2-(4-phenoxy-phenoxy)-ethoxy]-pyridine (pyriproxyfen; Sumitomo 31183)

**Supporting Table 2. Experimental versus CoMFA calculated biological activities (-log ED_50_) of**

**JH agonists.**

| **Compound** **Experimental** **Calculated** **Residual** |
| --- |
| **1**  1.42 1.72 -0.30 |
| **2**  –0.55 0.17 -0.72 |
| **3**  -0.76 0.41 -0.34 |
| **4**  -0.27 -0.49 0.22 |
| **5**  -0.72 -0.66 -0.06 |
| **6**  -0.95 -0.81 -0.14 |
| **7**  0.39 0.54 -0.15 |
| **8**  1.50 1.23 0.27 |
| **9**  3.69 3.31 0.38 |
| **10**  -1.30 -1.34 0.04 |
| **11**  1.06 1.17 -0.11 |
| **12**  -0.84 -0.92 0.08 |
| **13**  -0.92 -0.68 –0.24 |
| **14** 1.75 1.38 0.37 |
| **15** 2.46 1.72 0.74 |
| **16** 0.28 0.22 0.06 |
| **17** 2.39 1.46 0.93 |
| **18** -1.13 -0.95 -0.18 |
| **19** -1.23 -1.22 -0.01 |
| **20** 1.78 2.24 -0.46 |
| **21** 0.12 0.92 -0.80 |
| **22** -0.13 0.27 -0.40 |
| **23** -0.19 0.06 -0.25 |
| **24** -0.25 -0.51 0.26 |
| **25** -1.23 -1.09 -0.14 |
| **26** -0.71 -0.56 -0.15 |
| **27** -1.25 -1.19 -0.06 |
| **28** -0.45 -0.65 0.20 |
| **29** -1.60 -1.87 0.27 |
| **30** -1.61 -1.82 0.21 |
| **31** -1.58 -1.54 -0.04 |
| **32** -1.58 -1.46 -0.12 |
| **33** -1.55 -1.21 -0.34 |
| **34** -1.49 -1.48 -0.01 |
| **35** 1.84 1.77 0.06 |
| **36** 1.43 1.19 0.24 |
| **37** -1.21 -1.30 0.09 |
| **38** -1.53 -1.26 -0.27 |
| **39** -1.49 -1.47 -0.02 |
| **40** -1.15 -1.13 -0.02 |
| **41** -0.57 -0.45 -0.12 |
| **42** -1.12 -1.43 0.31 |
| **43** -0.71 -0.70 -0.01 |
| **44** -0.68 -0.32 -0.36 |
| **45** -1.27 -1.44 0.17 |
| **46** 1.76 1.75 0.01 |
| **47** -0.22 0.21 -0.43 |
| **48** -0.08 -0.39 0.31 |
| **49** -1.35 -1.30 -0.05 |
| **50** -0.14 -0.12 -0.02 |
| **51** -1.17 -1.14 -0.03 |
| **52** -1.15 -1.23 0.08 |
| **53** -1.07 -1.09 0.02 |
| **54** 1.45 1.10 0.35 |
| **55** 0.85 0.82 0.03 |
| **56** 2.38 2.50 -0.12 |
| **57** 0.78 0.47 0.31 |
| **58** 0.82 0.94 -0.12 |
| **59** -0.77 -0.74 -0.03 |
| **60** -0.42 -0.60 0.18 |
| **61** -1.25 -1.26 0.01 |
| **62** -0.39 -0.35 -0.04 |
| **63** -0.59 -0.62 0.03 |
| **64** -0.47 -0.43 -0.04 |
| **65** -1.04 -0.99 -0.05 |
| **66** -0.64 -0.40 -0.24 |
| **67** -0.49 -0.51 0.02 |
| **68** -0.64 -0.54 -0.10 |
| **69** 0.01 -0.38 0.39 |
| **70** -0.56 -0.79 0.23 |
| **71** -0.84 -0.84 0.00 |
| **72** -0.71 -1.09 0.38 |
| **73** -0.38 -0.16 -0.22 |
| **74** -0.61 -0.19 -0.42 |
| **75** -0.72 -0.81 0.09 |
| **76** -0.42 -0.37 -0.05 |
| **77** -0.88 -0.71 -0.17 |
| **78** 0.62 0.91 -0.29 |
| **79** -0.30 -0.31 0.01 |
| **80** -0.63 -0.25 -0.38 |
| **81** 2.20 1.88 0.32 |
| **82** 1.70 1.80 -0.10 |
| **83** 1.70 2.36 -0.66 |
| **84** 1.79 1.50 0.29 |
| **85** 1.77 1.61 0.16 |
| **86** 2.80 2.64 0.16 |

**Supporting Table 3. Test set Class I + II**

CoMFA predictive *r^2^* = 0.49

CoMSIA predictive *r^2^* = 0.51

| **Compound** **Experimental** **Calculated** **Residual** |
| --- |
| **13** -0.91 -1.38 -1.23 |
| **14** 1.75 1.37 1.48 |
| **18** -1.13 -0.76 -0.65 |
| **21** 0.12 0.31 0.24 |
| **31** -1.58 -1.33 -1.21 |
| **59** -1.34 -0.78 -0.89 |
| **63** -0.59 -0.32 -0.86 |
| **69** 0.01 -0.25 -0.21 |
| **76** -0.42 -0.72 -0.64 |
| **83** 1.70 2.12 1.93 |

**Supporting Table 4. Test set I**

CoMFA predictive *r^2^* = 0.54

CoMSIA predictive *r^2^* = 0.59

| **Compound** **Experimental** **Calculated** **Residual** |
| --- |
| **13** -0.91 -1.24 -1.12 |
| **14** 1.75 1.81 1.69 |
| **18** -1.13 -0.89 -0.96 |
| **21** 0.12 0.24 0.19 |
| **31** -1.58 -1.83 -1.36 |

**Supporting Table 5. Test set II**

CoMFA predictive *r^2^* = 0.60

CoMSIA predictive *r^2^* = 0.63

| **Compound** **Experimental** **Calculated** **Residual** |
| --- |
| **59** -1.34 -1.56 -1.49 |
| **63** -0.59 -0.47 -0.68 |
| **69** 0.01 -0.10 -0.05 |
| **76** -0.42 -0.65 -0.59 |
| **83** 1.70 1.89 1.83 |

**Supporting Figures**

**Supporting Figure 1**. **Comparative plot of experimental versus CoMFA predicted biological activities (-log ED_50_) of common training set (Class I + II) of 76 JH agonists**. Despite being structurally diverse, most active compounds in *Drosophila* share some common features, *i.e*. an electronegative atom (oxygen or nitrogen) at one end of the molecule and electronegative atom (epoxy oxygen) or electron rich moiety (oxyphenyl group) on the molecule‘s opposite end (see compounds **1** - **3**, **14** - **17**, **19**, **81** - **86**). Nonetheless, the terpenoid and rigid phenoxy structures have very different chemical reactivity, atom charges and abilities in forming hydrogen bonds or electrostatic interactions. Indeed, this was one major reason to divide the complete training set into two classes. The oxygen in phenoxyphenol group of Class II compounds is sterically hindered by benzene rings that makes the phenoxyphenol oxygen poorly reactive for intermolecular hydrogen bonding, while the oxygen within an epoxy moiety of Class I compounds can easily provide electron pairs for H-bonding or for other electrostatic interactions. The difference between Class I and II analogs is reflected also in their negative charge distribution. In the Class I structures it is concentrated near electronegative, ether or epoxy oxygen whereas in Class II structures it is localized to the phenyl rings. Indeed, a similar protocol for subdividing compounds into two chemotypes for QSAR analyses was published recently for COX-2 inhibitors [41] and steroid hormones to reflect the unusual conformational adaptation of nuclear receptor ligand binding domains to agonist variety [42, 43]. Furthermore, the presence of an electron deficient moiety in the middle of the JH agonist molecule is essential for the very high biological activity seen in some synthetic JH agonists but not observed in natural JH (blue and cyan polyhedra regions in CoMFA and CoMSIA contour maps, respectively; see Figures 2 and 3A, B). On the other hand, the steric CoMFA and CoMSIA contour maps indicate that presence of more bulky substituent in Class I compounds (green polyhedra) will enhance their biological activity. More bulky substituent (yellow polyhedra) near the phenoxyphenyl or epoxy groups in Class II compounds would decrease their biological activity (as again shown in Figures 2B and 3B). Thus, it is significant that each of these two independent training sets have shown a nearly ideal alignment and markedly better statistical parameters than the original common set.

**Supporting Figure 2**. **Graphical representation of observed versus CoMFA predicted biological activities (-log ED_50_) for training set of Class I JH agonists.**

**Supporting Figure 3**. **Correlation between experimental and CoMFA predicted biological activities (-log ED_50_) for training set of Class II JH agonists.**

**Supporting Figure 4**. **Graphical representation of observed versus CoMSIA predicted biological activities (-log ED_50_) for training set of Class I JH agonists**. The difference between Class I and Class II agonists in their hydrogen bonding availability is markedly visible in the CoMSIA hydrogen bond contour maps (for comparison see Figures 3C and 3D). There is a significant difference between Class I and Class II molecules in the large green area in the steric contour maps of both CoMSIA and the CoMFA (see also Supporting Figure 5). For Class I compounds the green area in this part of the structures is much smaller, and so it could signify a tighter contact with the receptor binding site.

**Supporting Figure 5**. **Experimental versus CoMSIA predicted biological activities (-log ED_50_) for training set of Class II JH agonists.** The significant difference between Class I and Class II molecules in the large green area in the steric contour maps of both CoMFA and the CoMSIA indicates that more bulky substituents in these regions will enhance the biological activity in Class II compounds. This might lead us to presume that there is a bigger or more flexible binding-site cavity surrounding this region in the agonists. The CoMSIA and also CoMFA generated steric and electrostatic contour maps have the potential to indicate the shape and surface requirements of the JH binding protein cavity, the putative JH-receptor. From this, we can infer that the receptor cavity must have charged residues lengthwise along its borders and negatively charged or neutral residues in its middle.

**Supporting References**

1. Ransom R (1982) A Handbook of Drosophila Development. Amsterdam and New York: Elsevier

Biomedical Press.

2. Ashburner M (1970) Effects of juvenile hormone on adult differentiation of *Drosophila*

*melanogaster*. Nature 227: 187-189.

3. Jarolím V, Hejno K, Sehnal F, Šorm F (1969) Natural and synthetic materials with insect

hormones activity. 8. Juvenile activity of the farnesane-type compounds on *Galleria* *mellonella*.

Life Sci 8: 831-841.

4. Benz G (1971) Failure to demonstrate sterilans effect of juvenile hormone mimetics in *Pieris*

*brassicae* and *Galleria mellonella*. Experientia 27: 581-582.

5. DeLoof A, Van de Veire M (1972) Time saving improvements in the *Galleria* bioassay for

juvenile hormone. Experientia 28: 366-367.

6. Sláma K, Romaňuk M, Šorm F (1974) Insect Hormones and Bioanalogues. Wien, Heidelberg

and New York: Springer Verlag.

7. Bowers ES (1969) Juvenile hormone: activity of aromatic terpenoid ethers. Science 164: 323-325.

8. Zaoral M, Sláma K (1970) Peptides with juvenile hormone activity. Science 170: 92-93.

9. Schwarz M, Redfern RE, Waters RM, Wakabayashi N, Sonnet PE (1971) Compounds related to

juvenile hormone. X. Activity of selected arylterpenoid compounds on *Tenebrio molitor* L. and

*Oncopeltus fasciatus* (Dallas). Life Sci II 10: 1125-1132.

10. Henrick CA, Staal GB, Siddall JB (1973) Alkyl 3,7,11-trimethyl-2,4-dodecadienoates, a new

class of potent insect growth regulators with juvenile hormone activity. J Agric Food Chem 21:

354-359.

11. Brieger G, Ellis RF (1975) Terpenoid ethers as juvenile hormone analogs. J Agric Food Chem

23: 335-337.

12. Henrick CA, Staal GB, Siddall JB (1973) Alkyl 3,7,11-trimethyl-2,4-dodecadienoates, a new

class of potent insect growth regulators with juvenile hormone activity. J Agric Food Chem

21: 354-359.

13. Henrick CA, Willy WE, Garcia BA, Staal GB (1975) Insect juvenile hormone activity of the

stereoisomers of ethyl 3,7,11-trimethyl-2,4-dodecadienoate. J Agric Food Chem 23: 396-400.

14. Henrick CA, Willy WE, Staal GB (1976) Insect juvenile hormone activity of alkyl (2E,4E)-

3,7,11-trimethyl-2,4-dodecadienoates. Variations in the ester function and in the carbon chain.

J Agric Food Chem 24: 207-218.

15. Sehnal F, Ždárek J (1976) Action of juvenoids on the metamorphosis of cyclorrhaphous diptera.

J Insect Physiol 22: 673-682.

16. Kramer KJ, McGregor HE, Mori K (1979) Susceptibility of stored product insects to pyridyl

ether analogues of juvenile hormone. J Agric Food Chem 27: 1215-1217.

17. Nakayama A, Iwamura H, Niwa A, Nakagawa Y, Fujita T (1985) Development of insect juvenile

hormone active oxime *o*-ethers and carbamates. J Agric Food Chem 33: 1034-1041.

18. Hatakoshi M, Agui N, Nakayama I (1986) 2-[1-Methyl-2-(4-phenoxy-phenoxy)ethoxy]-pyridine

as a new insect juvenile hormone analog induction of supernumerary larvae in *Spodoptera* *litura*

(*Lepidoptera*: *Noctuidae*). Appl Entomol Zool 21: 351-353.

19. Masner P, Angst M, Dorn S (1987) Fenoxycarb, an insect growth regulator with juvenile

hormone activity: A candidate for *Heliothis* *virescens* (F.) control on cotton. Pestic Sci 18: 89-94.

20. Niwa A, Iwamura H, Nakagawa Y, Fujita T (1989) Development of (phenoxyphenoxy)- and

(benzylphenoxy)-propyl ethers as potent insect juvenile hormone mimetics. J Agric Food Chem

37: 462-467.

21. Niwa A, Iwamura H, Nakagawa Y, Fujita T (1989) Development of (phenoxyphenoxy)- and

(benzylphenoxy)propyl ethers as potent insect juvenile hormone mimetics J Agric Food Chem

37: 467-472.

22. Henrick CA (1995) Juvenoids. In: Godfrey CRA, editor. Agrochemicals from Natural Products.

New York and Basel: Marcel Dekker Press. pp. 147-213.

23. Wimmer Z, Rejzek M, Zarevúcka M, Kuldová J, Hrdý I et al. (1997) A series of bicyclic insect

juvenile hormone analogs of Czech origin: Twenty years of development. J Chem Ecol 23: 605-628.

24. Maxwell RA, Anderson RJ, Schooley DA (2002) Simultaneous preparation of both enantiomers

of juvenile hormones labeled at C-10 with tritium at high specific activity. Anal Biochem 305:

40-48.

25. Ichikawa A, Ono H, Furuta K, Shiotsuki T, Shinoda T (2007) Enantioselective separation of

racemic juvenile hormone III by normal-phase high-performance liquid chromatography and

preparation of [^2^H_3_]juvenile hormone III as an internal standard for liquid chromatography-mass

spectrometry quantification. J Chromatogr A 1161: 252-260.

26. van der Meer JM (1977) Optical clean and permanent whole mount preparation for phase-contrast

microscopy of cuticular structures of insect. larvae. Dros Inf Serv 52: 160-161.

27. Yun B, Farkaš R, Lee K, Rabinow L (1994) The *Doa* locus encodes a member of a new protein

kinase family and is essential for eye and embryonic development in *Drosophila melanogaster*.

Genes Dev 8: 1160-1173.

28. Beňo M, Liszeková D, Farkaš R (2007) Processing of soft pupae and uneclosed pharate adults

of *Drosophila* for scanning electron microscopy. Micros Res Tech 70: 1022-1027.

29. Nation JL (1983) A new method using hexamethyldisilazane for preparation of soft insect tissues

for scanning electron microscopy. Stain Technol 58: 347-351.

30. Prestwich GD, Wawrzenczyk C (1985) High specific activity enantiomerically enriched juvenile

hormones: synthesis and binding assay. Proc Natl Acad Sci USA 82: 5290-5294.

31. Cody JT, Valtier S, Nelson SL (2004) Amphetamine excretion profile following multidose

administration of mixed salt amphetamine preparation. J Anal Toxicol 28: 563-574.

32. Gadler P, Faber K (2007) New enzymes for biotransformations: microbial alkyl sulfatases

displaying stereo- and enantioselectivity. Trends Biotechnol 25: 83-88.

33. Nillos MG, Rodriguez-Fuentes G, Gan J, Schlenk D (2007) Enantioselective

acetylcholinesterase inhibition of the organophosphorous insecticides profenofos, fonofos, and

crotoxyphos. Environ Toxicol Chem 26: 1949-1954.

34. Klaholz BP, Renaud JP, Mitschler A, Zusi C, Chambon P et al. (1998) Conformational

adaptation of agonists to the human nuclear receptor RAR gamma. Nature Struct Biol 5:

199-202.

35. Klaholz BP, Mitschler A, Belema M, Zusi C, Moras D (2000) Enantiomer discrimination

illustrated by high-resolution crystal structures of the human nuclear receptor hRARgamma.

Proc Natl Acad Sci USA 97: 6322-6327.

36. Klaholz BP, Mitschler A, Moras D (2000) Structural basis for isotype selectivity of the human

retinoic acid nuclear receptor. J Mol Biol 302: 155-170.

37. Golbraikh A, Bonchev D, Tropsha A (2001) Novel chirality descriptors derived from molecular

topology. J Chem Inf Comput Sci 41: 147-158.

38. Paier J, Stockner T, Steinreiber A, Faber K, Fabian WM (2003) Enantioselectivity of epoxide

hydrolase catalysed oxirane ring opening: a 3D QSAR study. J Comput Aided Mol Des 17: 1-11.

39. Kovatcheva A, Golbraikh A, Oloff S, Feng J, Zheng W et al. (2005) QSAR modeling of

datasets with enantioselective compounds using chirality sensitive molecular descriptors.

SAR QSAR Environ Res 16: 93-102.

40. Cramer RD, Patterson DE, Bunce JD (1989) Recent advances in comparative molecular field

analysis (CoMFA). Prog Clin Biol Res 291: 161-165.

41. Kim HJ, Chae CH, Yi KY, Park KL, Yoo SE (2004) Computational studies of COX-2

inhibitors: 3D-QSAR and docking. Biooorg Med Chem 12: 1629-1641.

42. Togashi M, Borngraeber S, Sandler B, Fletterick RJ, Webb P et al. (2005) Conformational

adaptation of nuclear receptor ligand binding domains to agonists: potential for novel approaches

to ligand design. J Steroid Biochem Mol Biol 93: 127-137.

43. Wheelock CE, Nakagawa Y, Harada T, Oikawa N, Akamatsu Met al. (2006) High-throughput

screening of ecdysone agonists using a reporter gene assay followed by 3-D QSAR analysis of the

molting hormonal activity. Biooorg Med Chem 14: 1143-1159.
