# Supplementary material for: Molecular Determinants of Juvenile Hormone Action as Revealed by 3D QSAR Analysis in Drosophila
Source: PLoS One. 2009 Jun 23;4(6):e6001. doi: 10.1371/journal.pone.0006001 (PMC2696086; doi:10.1371/journal.pone.0006001)
Supplement: Table S2 — (0.03 MB DOC) [file pone.0006001.s008.doc]

**Supporting Table 2. Experimental versus CoMFA calculated biological activities (-log ED_50_) of**

**JH agonists.**

| **Compound** **Experimental** **Calculated** **Residual** |
| --- |
| **1**  1.42 1.72 -0.30 |
| **2**  –0.55 0.17 -0.72 |
| **3**  -0.76 0.41 -0.34 |
| **4**  -0.27 -0.49 0.22 |
| **5**  -0.72 -0.66 -0.06 |
| **6**  -0.95 -0.81 -0.14 |
| **7**  0.39 0.54 -0.15 |
| **8**  1.50 1.23 0.27 |
| **9**  3.69 3.31 0.38 |
| **10**  -1.30 -1.34 0.04 |
| **11**  1.06 1.17 -0.11 |
| **12**  -0.84 -0.92 0.08 |
| **13**  -0.92 -0.68 –0.24 |
| **14** 1.75 1.38 0.37 |
| **15** 2.46 1.72 0.74 |
| **16** 0.28 0.22 0.06 |
| **17** 2.39 1.46 0.93 |
| **18** -1.13 -0.95 -0.18 |
| **19** -1.23 -1.22 -0.01 |
| **20** 1.78 2.24 -0.46 |
| **21** 0.12 0.92 -0.80 |
| **22** -0.13 0.27 -0.40 |
| **23** -0.19 0.06 -0.25 |
| **24** -0.25 -0.51 0.26 |
| **25** -1.23 -1.09 -0.14 |
| **26** -0.71 -0.56 -0.15 |
| **27** -1.25 -1.19 -0.06 |
| **28** -0.45 -0.65 0.20 |
| **29** -1.60 -1.87 0.27 |
| **30** -1.61 -1.82 0.21 |
| **31** -1.58 -1.54 -0.04 |
| **32** -1.58 -1.46 -0.12 |
| **33** -1.55 -1.21 -0.34 |
| **34** -1.49 -1.48 -0.01 |
| **35** 1.84 1.77 0.06 |
| **36** 1.43 1.19 0.24 |
| **37** -1.21 -1.30 0.09 |
| **38** -1.53 -1.26 -0.27 |
| **39** -1.49 -1.47 -0.02 |
| **40** -1.15 -1.13 -0.02 |
| **41** -0.57 -0.45 -0.12 |
| **42** -1.12 -1.43 0.31 |
| **43** -0.71 -0.70 -0.01 |
| **44** -0.68 -0.32 -0.36 |
| **45** -1.27 -1.44 0.17 |
| **46** 1.76 1.75 0.01 |
| **47** -0.22 0.21 -0.43 |
| **48** -0.08 -0.39 0.31 |
| **49** -1.35 -1.30 -0.05 |
| **50** -0.14 -0.12 -0.02 |
| **51** -1.17 -1.14 -0.03 |
| **52** -1.15 -1.23 0.08 |
| **53** -1.07 -1.09 0.02 |
| **54** 1.45 1.10 0.35 |
| **55** 0.85 0.82 0.03 |
| **56** 2.38 2.50 -0.12 |
| **57** 0.78 0.47 0.31 |
| **58** 0.82 0.94 -0.12 |
| **59** -0.77 -0.74 -0.03 |
| **60** -0.42 -0.60 0.18 |
| **61** -1.25 -1.26 0.01 |
| **62** -0.39 -0.35 -0.04 |
| **63** -0.59 -0.62 0.03 |
| **64** -0.47 -0.43 -0.04 |
| **65** -1.04 -0.99 -0.05 |
| **66** -0.64 -0.40 -0.24 |
| **67** -0.49 -0.51 0.02 |
| **68** -0.64 -0.54 -0.10 |
| **69** 0.01 -0.38 0.39 |
| **70** -0.56 -0.79 0.23 |
| **71** -0.84 -0.84 0.00 |
| **72** -0.71 -1.09 0.38 |
| **73** -0.38 -0.16 -0.22 |
| **74** -0.61 -0.19 -0.42 |
| **75** -0.72 -0.81 0.09 |
| **76** -0.42 -0.37 -0.05 |
| **77** -0.88 -0.71 -0.17 |
| **78** 0.62 0.91 -0.29 |
| **79** -0.30 -0.31 0.01 |
| **80** -0.63 -0.25 -0.38 |
| **81** 2.20 1.88 0.32 |
| **82** 1.70 1.80 -0.10 |
| **83** 1.70 2.36 -0.66 |
| **84** 1.79 1.50 0.29 |
| **85** 1.77 1.61 0.16 |
| **86** 2.80 2.64 0.16 |
