# Supplementary material for: Molecular Determinants of Juvenile Hormone Action as Revealed by 3D QSAR Analysis in Drosophila
Source: PLoS One. 2009 Jun 23;4(6):e6001. doi: 10.1371/journal.pone.0006001 (PMC2696086; doi:10.1371/journal.pone.0006001)
Supplement: Table S3 — (0.02 MB DOC) [file pone.0006001.s009.doc]

**Supporting Table 3. Test set Class I + II**

CoMFA predictive *r^2^* = 0.49

CoMSIA predictive *r^2^* = 0.51

| **Compound** **Experimental** **Calculated** **Residual** |
| --- |
| **13** -0.91 -1.38 -1.23 |
| **14** 1.75 1.37 1.48 |
| **18** -1.13 -0.76 -0.65 |
| **21** 0.12 0.31 0.24 |
| **31** -1.58 -1.33 -1.21 |
| **59** -1.34 -0.78 -0.89 |
| **63** -0.59 -0.32 -0.86 |
| **69** 0.01 -0.25 -0.21 |
| **76** -0.42 -0.72 -0.64 |
| **83** 1.70 2.12 1.93 |
