# Supplementary material for: Molecular Determinants of Juvenile Hormone Action as Revealed by 3D QSAR Analysis in Drosophila
Source: PLoS One. 2009 Jun 23;4(6):e6001. doi: 10.1371/journal.pone.0006001 (PMC2696086; doi:10.1371/journal.pone.0006001)
Supplement: Table S4 — (0.02 MB DOC) [file pone.0006001.s010.doc]

**Supporting Table 4. Test set I**

CoMFA predictive *r^2^* = 0.54

CoMSIA predictive *r^2^* = 0.59

| **Compound** **Experimental** **Calculated** **Residual** |
| --- |
| **13** -0.91 -1.24 -1.12 |
| **14** 1.75 1.81 1.69 |
| **18** -1.13 -0.89 -0.96 |
| **21** 0.12 0.24 0.19 |
| **31** -1.58 -1.83 -1.36 |
