# Supplementary material for: Molecular Determinants of Juvenile Hormone Action as Revealed by 3D QSAR Analysis in Drosophila
Source: PLoS One. 2009 Jun 23;4(6):e6001. doi: 10.1371/journal.pone.0006001 (PMC2696086; doi:10.1371/journal.pone.0006001)
Supplement: Table S5 — (0.02 MB DOC) [file pone.0006001.s011.doc]

**Supporting Table 5. Test set II**

CoMFA predictive *r^2^* = 0.60

CoMSIA predictive *r^2^* = 0.63

| **Compound** **Experimental** **Calculated** **Residual** |
| --- |
| **59** -1.34 -1.56 -1.49 |
| **63** -0.59 -0.47 -0.68 |
| **69** 0.01 -0.10 -0.05 |
| **76** -0.42 -0.65 -0.59 |
| **83** 1.70 1.89 1.83 |
