# Supplementary material for: Molecular Determinants of Juvenile Hormone Action as Revealed by 3D QSAR Analysis in Drosophila
Source: PLoS One. 2009 Jun 23;4(6):e6001. doi: 10.1371/journal.pone.0006001 (PMC2696086; doi:10.1371/journal.pone.0006001)
Supplement: References S1 — (0.04 MB DOC) [file pone.0006001.s012.doc]

# Supporting Information

**Supporting References**

1. Ransom R (1982) A Handbook of Drosophila Development. Amsterdam and New York: Elsevier

Biomedical Press.

2. Ashburner M (1970) Effects of juvenile hormone on adult differentiation of *Drosophila*

*melanogaster*. Nature 227: 187-189.

3. Jarolím V, Hejno K, Sehnal F, Šorm F (1969) Natural and synthetic materials with insect

hormones activity. 8. Juvenile activity of the farnesane-type compounds on *Galleria* *mellonella*.

Life Sci 8: 831-841.

4. Benz G (1971) Failure to demonstrate sterilans effect of juvenile hormone mimetics in *Pieris*

*brassicae* and *Galleria mellonella*. Experientia 27: 581-582.

5. DeLoof A, Van de Veire M (1972) Time saving improvements in the *Galleria* bioassay for

juvenile hormone. Experientia 28: 366-367.

6. Sláma K, Romaňuk M, Šorm F (1974) Insect Hormones and Bioanalogues. Wien, Heidelberg

and New York: Springer Verlag.

7. Bowers ES (1969) Juvenile hormone: activity of aromatic terpenoid ethers. Science 164: 323-325.

8. Zaoral M, Sláma K (1970) Peptides with juvenile hormone activity. Science 170: 92-93.

9. Schwarz M, Redfern RE, Waters RM, Wakabayashi N, Sonnet PE (1971) Compounds related to

juvenile hormone. X. Activity of selected arylterpenoid compounds on *Tenebrio molitor* L. and

*Oncopeltus fasciatus* (Dallas). Life Sci II 10: 1125-1132.

10. Henrick CA, Staal GB, Siddall JB (1973) Alkyl 3,7,11-trimethyl-2,4-dodecadienoates, a new

class of potent insect growth regulators with juvenile hormone activity. J Agric Food Chem 21:

354-359.

11. Brieger G, Ellis RF (1975) Terpenoid ethers as juvenile hormone analogs. J Agric Food Chem

23: 335-337.

12. Henrick CA, Staal GB, Siddall JB (1973) Alkyl 3,7,11-trimethyl-2,4-dodecadienoates, a new

class of potent insect growth regulators with juvenile hormone activity. J Agric Food Chem

21: 354-359.

13. Henrick CA, Willy WE, Garcia BA, Staal GB (1975) Insect juvenile hormone activity of the

stereoisomers of ethyl 3,7,11-trimethyl-2,4-dodecadienoate. J Agric Food Chem 23: 396-400.

14. Henrick CA, Willy WE, Staal GB (1976) Insect juvenile hormone activity of alkyl (2E,4E)-

3,7,11-trimethyl-2,4-dodecadienoates. Variations in the ester function and in the carbon chain.

J Agric Food Chem 24: 207-218.

15. Sehnal F, Ždárek J (1976) Action of juvenoids on the metamorphosis of cyclorrhaphous diptera.

J Insect Physiol 22: 673-682.

16. Kramer KJ, McGregor HE, Mori K (1979) Susceptibility of stored product insects to pyridyl

ether analogues of juvenile hormone. J Agric Food Chem 27: 1215-1217.

17. Nakayama A, Iwamura H, Niwa A, Nakagawa Y, Fujita T (1985) Development of insect juvenile

hormone active oxime *o*-ethers and carbamates. J Agric Food Chem 33: 1034-1041.

18. Hatakoshi M, Agui N, Nakayama I (1986) 2-[1-Methyl-2-(4-phenoxy-phenoxy)ethoxy]-pyridine

as a new insect juvenile hormone analog induction of supernumerary larvae in *Spodoptera* *litura*

(*Lepidoptera*: *Noctuidae*). Appl Entomol Zool 21: 351-353.

19. Masner P, Angst M, Dorn S (1987) Fenoxycarb, an insect growth regulator with juvenile

hormone activity: A candidate for *Heliothis* *virescens* (F.) control on cotton. Pestic Sci 18: 89-94.

20. Niwa A, Iwamura H, Nakagawa Y, Fujita T (1989) Development of (phenoxyphenoxy)- and

(benzylphenoxy)-propyl ethers as potent insect juvenile hormone mimetics. J Agric Food Chem

37: 462-467.

21. Niwa A, Iwamura H, Nakagawa Y, Fujita T (1989) Development of (phenoxyphenoxy)- and

(benzylphenoxy)propyl ethers as potent insect juvenile hormone mimetics J Agric Food Chem

37: 467-472.

22. Henrick CA (1995) Juvenoids. In: Godfrey CRA, editor. Agrochemicals from Natural Products.

New York and Basel: Marcel Dekker Press. pp. 147-213.

23. Wimmer Z, Rejzek M, Zarevúcka M, Kuldová J, Hrdý I et al. (1997) A series of bicyclic insect

juvenile hormone analogs of Czech origin: Twenty years of development. J Chem Ecol 23: 605-628.

24. Maxwell RA, Anderson RJ, Schooley DA (2002) Simultaneous preparation of both enantiomers

of juvenile hormones labeled at C-10 with tritium at high specific activity. Anal Biochem 305:

40-48.

25. Ichikawa A, Ono H, Furuta K, Shiotsuki T, Shinoda T (2007) Enantioselective separation of

racemic juvenile hormone III by normal-phase high-performance liquid chromatography and

preparation of [^2^H_3_]juvenile hormone III as an internal standard for liquid chromatography-mass

spectrometry quantification. J Chromatogr A 1161: 252-260.

26. van der Meer JM (1977) Optical clean and permanent whole mount preparation for phase-contrast

microscopy of cuticular structures of insect. larvae. Dros Inf Serv 52: 160-161.

27. Yun B, Farkaš R, Lee K, Rabinow L (1994) The *Doa* locus encodes a member of a new protein

kinase family and is essential for eye and embryonic development in *Drosophila melanogaster*.

Genes Dev 8: 1160-1173.

28. Beňo M, Liszeková D, Farkaš R (2007) Processing of soft pupae and uneclosed pharate adults

of *Drosophila* for scanning electron microscopy. Micros Res Tech 70: 1022-1027.

29. Nation JL (1983) A new method using hexamethyldisilazane for preparation of soft insect tissues

for scanning electron microscopy. Stain Technol 58: 347-351.

30. Prestwich GD, Wawrzenczyk C (1985) High specific activity enantiomerically enriched juvenile

hormones: synthesis and binding assay. Proc Natl Acad Sci USA 82: 5290-5294.

31. Cody JT, Valtier S, Nelson SL (2004) Amphetamine excretion profile following multidose

administration of mixed salt amphetamine preparation. J Anal Toxicol 28: 563-574.

32. Gadler P, Faber K (2007) New enzymes for biotransformations: microbial alkyl sulfatases

displaying stereo- and enantioselectivity. Trends Biotechnol 25: 83-88.

33. Nillos MG, Rodriguez-Fuentes G, Gan J, Schlenk D (2007) Enantioselective

acetylcholinesterase inhibition of the organophosphorous insecticides profenofos, fonofos, and

crotoxyphos. Environ Toxicol Chem 26: 1949-1954.

34. Klaholz BP, Renaud JP, Mitschler A, Zusi C, Chambon P et al. (1998) Conformational

adaptation of agonists to the human nuclear receptor RAR gamma. Nature Struct Biol 5:

199-202.

35. Klaholz BP, Mitschler A, Belema M, Zusi C, Moras D (2000) Enantiomer discrimination

illustrated by high-resolution crystal structures of the human nuclear receptor hRARgamma.

Proc Natl Acad Sci USA 97: 6322-6327.

36. Klaholz BP, Mitschler A, Moras D (2000) Structural basis for isotype selectivity of the human

retinoic acid nuclear receptor. J Mol Biol 302: 155-170.

37. Golbraikh A, Bonchev D, Tropsha A (2001) Novel chirality descriptors derived from molecular

topology. J Chem Inf Comput Sci 41: 147-158.

38. Paier J, Stockner T, Steinreiber A, Faber K, Fabian WM (2003) Enantioselectivity of epoxide

hydrolase catalysed oxirane ring opening: a 3D QSAR study. J Comput Aided Mol Des 17: 1-11.

39. Kovatcheva A, Golbraikh A, Oloff S, Feng J, Zheng W et al. (2005) QSAR modeling of

datasets with enantioselective compounds using chirality sensitive molecular descriptors.

SAR QSAR Environ Res 16: 93-102.

40. Cramer RD, Patterson DE, Bunce JD (1989) Recent advances in comparative molecular field

analysis (CoMFA). Prog Clin Biol Res 291: 161-165.

41. Kim HJ, Chae CH, Yi KY, Park KL, Yoo SE (2004) Computational studies of COX-2

inhibitors: 3D-QSAR and docking. Biooorg Med Chem 12: 1629-1641.

42. Togashi M, Borngraeber S, Sandler B, Fletterick RJ, Webb P et al. (2005) Conformational

adaptation of nuclear receptor ligand binding domains to agonists: potential for novel approaches

to ligand design. J Steroid Biochem Mol Biol 93: 127-137.

43. Wheelock CE, Nakagawa Y, Harada T, Oikawa N, Akamatsu Met al. (2006) High-throughput

screening of ecdysone agonists using a reporter gene assay followed by 3-D QSAR analysis of the

molting hormonal activity. Biooorg Med Chem 14: 1143-1159.
